# Regulatory changes underlying the evolution of skylight navigation

**DOI:** 10.1016/j.isci.2026.116313

**Published:** 2026-06-11

**Authors:** Heidi Roth, Melanie Sarfert, Aleksandra Simdianova, Jana Balke, Michael W. Perry, Katja Nowick, Mathias F. Wernet

**Affiliations:** 1 Freie Universität, Fachbereich Biologie, Chemie und Pharmazie, Institut für Biologie – Abteilung Neurobiologie, Königin-Luise Strasse 1-3, Berlin 14195, Germany; 2 Freie Universität, Fachbereich Biologie, Chemie and Pharmazie, Institut für Biologie – Abteilung Zoologie, Königin-Luise Strasse 1-3, Berlin 14195, Germany; 3 Department of Cell and Developmental Biology, School of Biological Sciences, University of California, San Diego, La Jolla, San Diego, CA 92093, USA

**Keywords:** Zoology, Entomology, Genetics, Evolutionary biology

## Abstract

Polarized skylight is an ancient navigational cue for many animals. In insects, its detection relies on the dorsal rim area (DRA), a specialized region of the compound eye. DRAs are widespread and vary in morphology and spectral tuning, raising the question of how this diversity evolved. Here we show that the DRA expression of the *Drosophila* transcription factor homothorax (encoded by the gene *hth*) depends on a 675 bp minimal enhancer. Genome editing of this enhancer abolishes both DRA formation and polarization-guided behavior. Furthermore, mutagenesis of conserved motifs suggests that enhancer activity is regulated by cooperative transcription factor activity. Newly generated antibodies from honeybees, butterflies, and mosquitoes reveal no *hth* expression in their DRAs, whereas *hth* is expressed across higher flies (Brachycera), coinciding with the loss of its homolog Pknox/Prep1. Phylogenetic comparisons therefore suggest that *hth*'s role in DRA specification arose relatively recently, coinciding with evolutionary changes in photoreceptor optics and photochemistry from lower to higher flies.

## Highlights

• Minimal *homothorax* enhancer for skylight polarization circuitry and behavior in *Drosophila*
• Minimal *hth* enhancer expression is regulated by cooperative transcription factor activity
• *hth* function is limited to higher flies, mirroring photoreceptor morphology and physiology

## Graphical abstract

**Figure:**
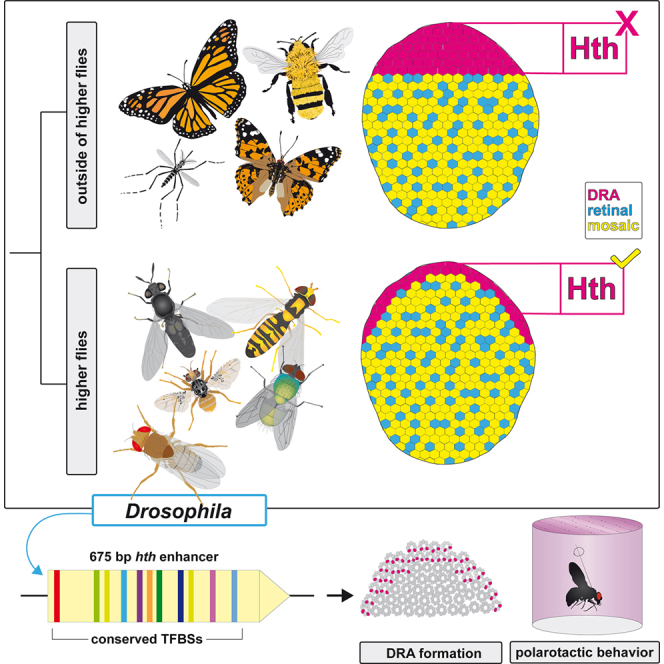

## Introduction

**Figure 1:**
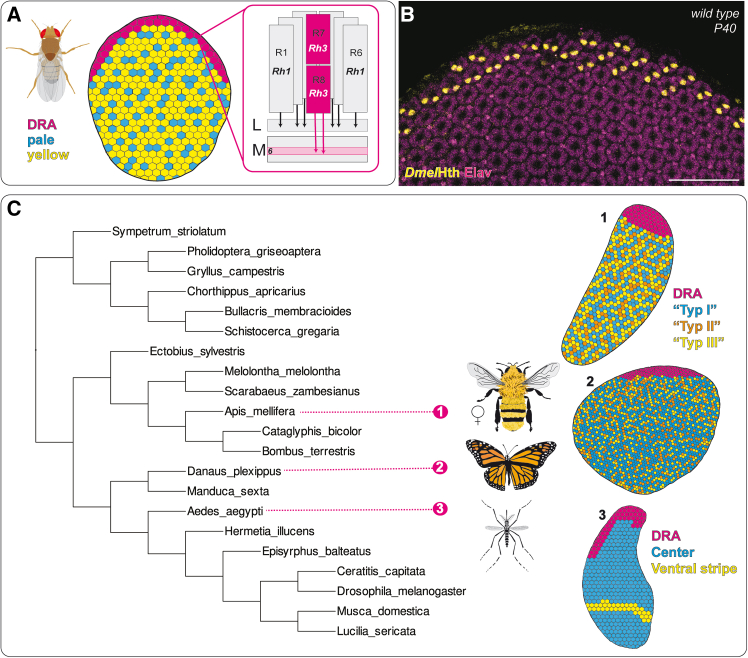
Retinal organization and DRA specification across insects (A) Schematic representation of *Drosophila* retinal mosaic highlights dorsal rim area (DRA) ommatidia (magenta), where both R7 and R8 photoreceptors express Rh3 and terminate in the same medulla layer (M6; adapted from 9). Pale and yellow ommatidia, which mediate color vision, are stochastically distributed across the retina. (B) Immunolabeling of *hth* expression in DRA R7 and R8 photoreceptors at pupal stage P40 (yellow). All photoreceptors are labeled with Elav (magenta). (C) Phylogenetic tree of insect taxa with characterized DRAs, highlighting species analyzed in this study (adapted after 8). Illustrations represent retinal mosaics of honeybee (1), worker, butterfly (2), and mosquito (3), emphasizing their distinct ommatidial organizations (adapted from 2).

Insects have evolved highly specialized, fast, and efficient visual systems, which are all based on variations of the compound eye, a conserved organizational principle across invertebrates.^1^^,^^2^ Each compound eye consists of numerous functional units, called ommatidia, that collectively sample visual space as discrete pixels. Depending on species and ecological niche, visual resolution and sensitivity vary with optical design (apposition vs. superposition), facet number, and size.^3^ Each ommatidium contains a stereotyped set of photoreceptors whose rhabdomeres contain rhodopsin molecules, as well as the molecular machinery required for phototransduction.^4^ In most insect species, “fused rhabdoms” integrate the microvilli of neighboring photoreceptors within each ommatidium,^5^^,^^6^^,^^7^ whereas higher dipterans such as *Drosophila* possess a neural superposition eye, in which the rhabdomeres of all eight photoreceptors (R1-8) within one ommatidium remain optically separated Figure 1A).^5^^,^^8^ This unique optical arrangement, in turn, necessitated the evolution of a complex neural wiring scheme that preserves spatial mapping by converging inputs from neighboring ommatidia onto shared postsynaptic targets.^5^

Across insect species, ommatidia differ by rhodopsin expression, rhabdomere morphology, and projection patterns to the optic lobes.^9^ Despite their vast morphological differences, the molecular specification of insect photoreceptors appears to be conserved, involving transcriptional regulators such as Spalt (*salm,* FBgn0261648), Defective proventriculus (*dve,* FBgn0020307), Bar (*Bar*, FBgn0000154), and Prospero (*pros*, FBgn0004595).^2^ In many cases, photoreceptors with distinct spectral sensitivities form stochastic mosaics supporting color vision. In *Drosophila*, this mosaic arises from the random expression of the transcription factor Spineless (*ss,* FBgn0003513) in R7 photoreceptors (ON/OFF) (Figure 1A).^10^ Remarkably, the same stochastic mechanism involving Spineless is conserved in butterflies, where the duplication of R7-like cells generates three ommatidial types (ON/ON, ON/OFF, and OFF/OFF) and expanded color opponency (Figure 1C).^11^ Thus, a shared molecular mechanism has yielded distinct retinal mosaics across insect species, suited to their visual demands.

Virtually all insect eyes include a regionalized subset of ommatidia at the dorsal margin, the dorsal rim area (DRA), dedicated to detecting linearly polarized skylight (Figure 1C).^8^^,^^12^^,^^13^ In *Drosophila*, polarization-sensitive inner photoreceptors in the DRA are homochromatic rhabdomeres of enlarged cross-sectional areas, enhancing sensitivity to polarized light (Figure 1A). A hallmark of DRA architecture is the orthogonal and untwisted arrangement of microvilli between two groups of photoreceptors (R7/R8 in flies), enabling antagonistic comparison of orthogonal angle of polarization (AoP) and thus polarization opponency.^8^^,^^13^ The spatial extent of the DRA varies widely across insects: Most species exhibit a rather restricted dorsal patch of ommatidia, whereas the DRA of higher flies (Brachycera) consists of a narrow band along the eye's dorsal rim (Figures 1A and 1C). The ethological significance of these structural differences remains unresolved.

Whether all insect DRAs derive from a common ancestor or represent convergent evolutionary solutions to similar navigational challenges remains unknown. In *Drosophila*, the specification of DRA ommatidia requires the TALE-class homeodomain (HD) transcription factor Homothorax (*hth,* FBgn0001235), expressed in developing R7 and R8 photoreceptors of the DRA throughout adulthood, as well as in the pigment rim surrounding the developing retina (Figure 1B).^14^^,^^15^^,^^16^ Loss- and gain-of-function studies established *hth* as the master regulator of DRA fate, acting in concert with transcription factors such as Spalt, Optomotor-blind *(*encoded by the gene *bi*, FBgn0000179,^16^ and dorsal selector genes of the *Iroquois* complex (*Iro-C*, comprising the genes *mirr*, *ara*, and *caup*).^17^ DRA development is induced by the diffusible morphogen Wingless (*wg*, FBgn0284084,^16^^,^^17^^,^^18^ whose role in retinal patterning is conserved across insects.^19^ At the molecular level, Hth depends on its obligatory co-factor Extradenticle *(exd,* FBgn0000611), which translocates to the nucleus in an Hth-dependent manner.^16^^,^^20^^,^^21^ Hth and Exd proteins are homologs of vertebrate Meis and Pbx, evolutionarily conserved cofactors of Hox*-*dependent developmental regulation.

Here, we combined immunohistochemistry, molecular genetics, behavior experiments, and bioinformatics to investigate the possible evolutionary conservation of the *hth/exd* mechanism in DRA specification across insect species. In *Drosophila,* we first identified a 675 bp minimal enhancer within the *hth* locus, sufficient to drive DRA-specific expression in developing R7 and R8 photoreceptors. CRISPR/Cas9 deletion of this enhancer abolished DRA-specific features and disrupted polarization-dependent navigation. Bioinformatic analysis and site-directed mutagenesis further revealed conserved transcription factor binding motifs (TFBM) that define a molecular fingerprint for DRA specification in flies. Newly generated antibodies against the predicted Hth proteins of honeybees (*Apis mellifera*), Monarch butterflies (*Danaus plexippus*) and mosquitoes (*Aedes aegypti*), revealed that *hth* expression is not conserved in developing DRA photoreceptors of these species, although pigment rim expression persisted. Interestingly, the genomes of all species except higher flies contain a gene encoding a homolog of Hth, a transcription factor named Pknox/Prep1, which can translocate *Exd* to the nucleus. This observation raises the possibility that during the evolution of higher flies, when open rhabdoms and altered optical properties emerged, *pknox/prep 1* was lost, and *hth* may have been recruited to assume a central role in DRA specification, resulting in a change of the regulatory architecture underlying DRA development.

## Results

### The minimal *hth* enhancer regulating DRA photoreceptor expression

In the past, the transcription factor Hth has been established as a master regulator of DRA specification in *Drosophila melanogaster*, regulated by *Iro-C* dorsal selector genes and the *wg* pathway.^18^ In order to investigate a potential evolutionary conservation of Hth’s role, we therefore sought to better understand how *hth* expression is transcriptionally regulated within the developing *Drosophila* DRA. The *hth* locus of *Drosophila* resides on the right arm of chromosome 3, spanning approximately 100 kb, and producing six distinct isoforms through alternative splicing (JBrowse (flybase.org),.^22^ Its pre-mRNA consists of 14 short exons, with exons 2–6 encoding the HM domain necessary for interaction with Hth’s mandatory co-factor Exd,^23^^,^^24^ and exons 11–13 encoding the DNA-binding HD.^25^ Among the mature transcripts, two isoforms (HthRA, HthRC) contain both domains, while three encode only the HD domain (HthRE, HthRF, HthRI). The expression of these isoforms differs both in time and space during development. The overall architecture of the *hth* locus is highly conserved across species.^26^

**Figure 2:**
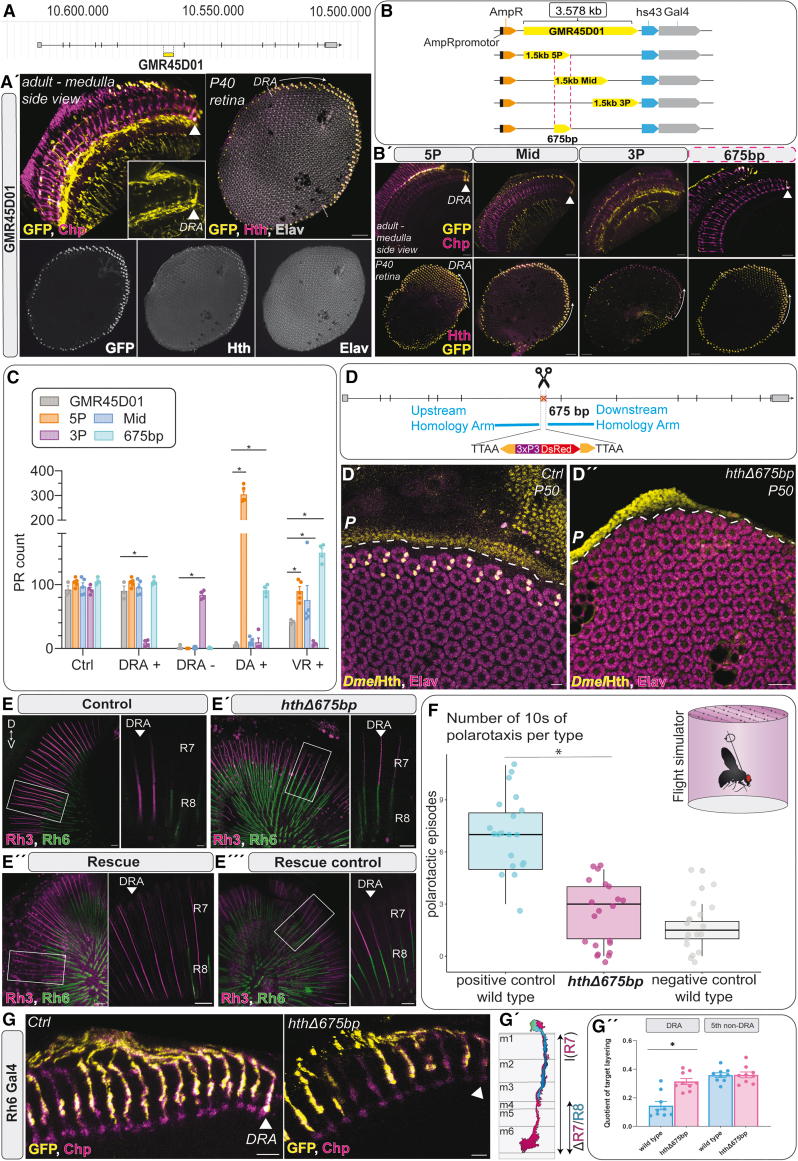
Identification and functional validation of a minimal DRA-specific *hth* enhancer (A) The hth locus showing the original GMR45D01 enhancer located within intron 6 of the HthRC isoform (adapted from FlyBase.org). (A′) GMR45D01-Gal4 drives expression in DRA R7/8 photoreceptors (adult, pupae) and rim cells, matching Anti-Hth antibody labeling (in pupae). (B) Design of DRA-specific Gal4 constructs: the original 3.5 kb GMR45D01-Gal4 was divided into three overlapping 1.5 kb fragments (5P, Mid, 3P), with the 5P-Mid overlap generating a 675 bp minimal enhancer. (B′) Adult expression: 5P, Mid, and 675bp enhancers drive GFP in DRA photoreceptors, while 3P labels medulla/lobula neurons. Pupal expression: 5P and Mid label DRA and ventral rim (VR) photoreceptors, with 5P also active in dorsal area (DA) R7/R8; 3P labels few equatorial PRs; 675 bp shows a combined 5P/Mid pattern with reduced DA labeling. (C) Quantification of pupal expression patterns (from B′). All enhancers except 3P label DRA R7/R8. 5P and 675bp show expanded DA and VR expression. Unpaired *t* test with Welch correction; *p* < 0.05 = ∗. *N* = 5 per line (except GMR45D01, *N* = 3). Error bars: mean ± SEM. (D) Schematic of CRISPR/Cas9-mediated deletion of the 675bp enhancer (genomic coordinates: 3R: 10578025–10578699), replaced by PBacDsRed marker cassette. (D′ and D'') P50 pupal retinas from control (wild type) and *hthΔ675bp* mutants stained with Anti-Hth antibody. In mutants, *hth* expression is lost in DRA R7/R8 but retained in the pigment rim (“P”). *N* = 6 (Ctrl), *N* = 7 (*hthΔ675bp*). (E) Rhodopsin staining in adult retinas. Wild-type DRA R7/R8 expresses Rh3; outside the DRA, R8 expresses Rh6, and R7 expresses Rh3 or remains unstained. Boxed regions highlight the DRA/non-DRA boundary. (E′) In *hthΔ675bp* mutants, dorsal R8 cells predominantly express Rh6 instead of Rh3. (E'') Reintroduction of Hth (UAS-Hth; driven by GMR45D01-Gal4) restores DRA-specific Rh3 expression in R7/R8. (E''') Negative control shows the mutant-like mismatched Rh3/Rh6 pairing. (F) Behavioral assay for polarized-light detection using a tethered-flight simulator (illustrated in scheme adapted from 31, 32). Wild-type flies track rotating e-vectors (positive control), whereas negative controls (polarizer swapped to block polarized light) show fewer polarotactic episodes. *hthΔ675bp* mutants behave like negative controls, showing severely reduced polarotaxis. Wilcoxon signed-rank test, *p* < 0.05 = ∗. *N* = 20. Error bars: mean ± SEM. (G) Analysis of R8 axon targeting using Rh6-Gal4, which weakly labels DRA R8. In wild type, DRA R8 axons project to M6, while non-DRA R8 axons target M3. In *hthΔ675bp* mutants, DRA R8 axons terminate more superficially but not fully in non-DRA positions. (G′) Quantification schematic (after 68). (G'') Targeting ratios show that *hthΔ675bp* DRA R8 terminals terminate significantly more superficially than wild type (*p* < 0.05 = ∗), while non-DRA R8 targeting remains unchanged. *N* = 9. Scale bars, 50 μm (medulla), 20 μm (retina).

We first aimed to identify the minimal enhancer sequence necessary and sufficient to drive *hth* expression in R7 and R8 photoreceptors of the *Drosophila* DRA. Screening of all first-generation Gal4 enhancer lines spanning the *hth* locus^27^^,^^28^ revealed a 3.5 kb fragment (GMR45D01) that drove strong and specific expression in DRA photoreceptors of adult flies, located within intron 6 of both long HthRA and HthRC isoforms as well as 3 prime of the three short isoforms HthRE, HthRF, HthRI (Figure 2A and A′). Further expression analysis in pupal retinas confirmed that this enhancer recapitulated endogenous Hth expression in DRA R7 and R8 cells (±42 R7/R8) (Figure 2A'). To define the minimal regulatory sequence within GMR45D01, we subdivided the 3.5 kb fragment into three overlapping 1.5 kb subfragments (termed 5P, Mid, and 3P; Figure 2B). Both 5P and Mid fragments retained DRA-specific expression, while 3P labeled only a subset of medulla and lobula neurons and lacked DRA specificity (Figure 2B'). We then generated a 675 bp fragment corresponding to the overlap between 5P and Mid. This 675 bp region also largely reproduced the DRA-specific pattern observed for 5P and Mid, driving expression predominantly in DRA R7 and R8 photoreceptors (Figure 2B'). Quantification of expression using Anti-Hth as counterstain revealed that this minimal enhancer maintained robust DRA expression, with some expansion into the dorsal area (DA) adjacent to the DRA, as well as the ventral rim (VR), at levels comparable to the full-length enhancer (Figure 2C). Despite this relatively mild non-DRA expression (±90 R7/R8, when compared to the total number of R7 and R8), the 675 bp fragment was sufficient to robustly drive Hth expression in the endogenous DRA. Hence, due to its small size, the 675 bp enhancer element was chosen as a platform for identifying potential regulatory motifs critical for directing DRA photoreceptor specification.

We next decided to test whether the identified minimal enhancer is indeed necessary for DRA fate specification *in vivo*, aiming for a CRISPR/Cas9 deletion of the 675 bp sequence. We first tested whether this sequence drives *hth* expression in other developing tissues, since broad expression across tissues and developmental stages would suggest a broader developmental role, leading to lethality upon deletion. No 675 bp enhancer-driven GFP reporter expression was detected in eye-antennal, wing, or leg discs, and only sparse labeling was observed in a few cells of the larval ventral nerve cord (*SI Appendix*, Figure S2). Hence, these results indicated that the pupal expression of the minimal *hth* enhancer is largely DRA-specific and therefore unlikely to regulate essential developmental processes outside the developing retina. We then proceeded to delete the 675 bp sequence from the endogenous *hth* locus using CRISPR/Cas9 genome editing (Figure 2D). In accordance with our expression analysis, homozygous deletion mutants (*hthΔ675bp*) were viable and displayed normal external morphology. However, analysis of P50 pupal retinas revealed a complete loss of Hth protein expression in DRA R7 and R8 photoreceptors, while a strong Anti-Hth signal remained in retinal pigment cells, as well as the surrounding pigment rim (Figure 2D' and D'').

We concluded that the 675 bp minimal enhancer functions as the sole DRA-specific enhancer of *hth*, with no evidence for redundant “shadow” enhancers.^29^^,^^30^ Since *hth* is both necessary and sufficient for inducing the *Drosophila* DRA fate, we next examined whether loss of the 675 bp minimal enhancer disrupts known hallmark DRA-specific structural and functional features: rhodopsin expression, axon layer targeting, and polarotactic behavior. In wild type flies, both R7 and R8 cells within the DRA express the UV opsin Rh3, whereas non-DRA R8 cells express Rh6 in yellow (65%), or Rh5 in pale ommatidia (35%) (Figures 2E and S3). In *hthΔ675 bp* mutants, DRA R7 cells retained Rh3 expression, while underlying R8 cells expressed Rh6. This “odd-coupled” expression of Rh3/Rh6 within the same ommatidium therefore reproduced the ommatidial phenotype previously observed upon genetically induced loss of *hth* function in photoreceptors (Figure 2E*'*, 15). This loss-of-function phenotype was reversed by reintroducing *hth* specifically into DRA R7 and R8 photoreceptors, using the GMR45D01-Gal4 driver to express a UAS-Hth transgene in an otherwise homozygous *hthΔ675 bp* background. In this genetic background, normal DRA-type Rh3 expression in both R7 and R8 at the dorsal rim of the adult retina was restored, and Rh6 was absent from rim R8 (Figure 2E''). We next analyzed axonal targeting of DRA photoreceptors into the distal medulla neuropil. In wild-type flies, only DRA R8 axons terminate in the deep M6 layer together with R7, whereas non-DRA R8 axons always project to the more distal M3 layer. Indeed, DRA R8 axons of homozygous *hthΔ675 bp* mutants terminated in significantly more superficial medulla layers (Figure 2G). Quantitative analysis of axonal depth confirmed this shift into layer M3, while non-DRA R8 projections remained unchanged (Figure 2G''). Thus, based on all available markers, the deletion of the 675bp enhancer converts DRA cells into non-DRA-like photoreceptors both at the molecular and circuit levels. Finally, we assessed whether the observed loss of DRA identity ultimately affects behavioral responses to linearly polarized light. Using a virtual flight simulator exposing magnetically tethered single flies to linearly polarized UV illumination (adapted after 31, 32), wild type flies adjusted their heading to slowly rotating stimulus, while flies exposed to unpolarized UV light did not (Figure 2F). In contrast, *hthΔ675 bp* mutants failed to adjust their heading under polarized light, resembling the unpolarized control condition, again confirming that the DRA is necessary for mediating polarotactic responses.^31^

Together, these findings establish a 675 bp enhancer within exon 6 of the *hth* locus as being both necessary and sufficient for *hth* expression in developing DRA inner photoreceptors. Its deletion eliminates DRA specification, disrupting rhodopsin expression, axonal targeting, and abolishing behavioral responses to linearly polarized. This phenotype closely mirrors the *hth* loss-of-function, providing a direct causal link between the minimal *hth* enhancer activity and DRA specialization and function.

### Conserved transcription factor binding sites act cooperatively to regulate the minimal DRA enhancer

**Figure 3:**
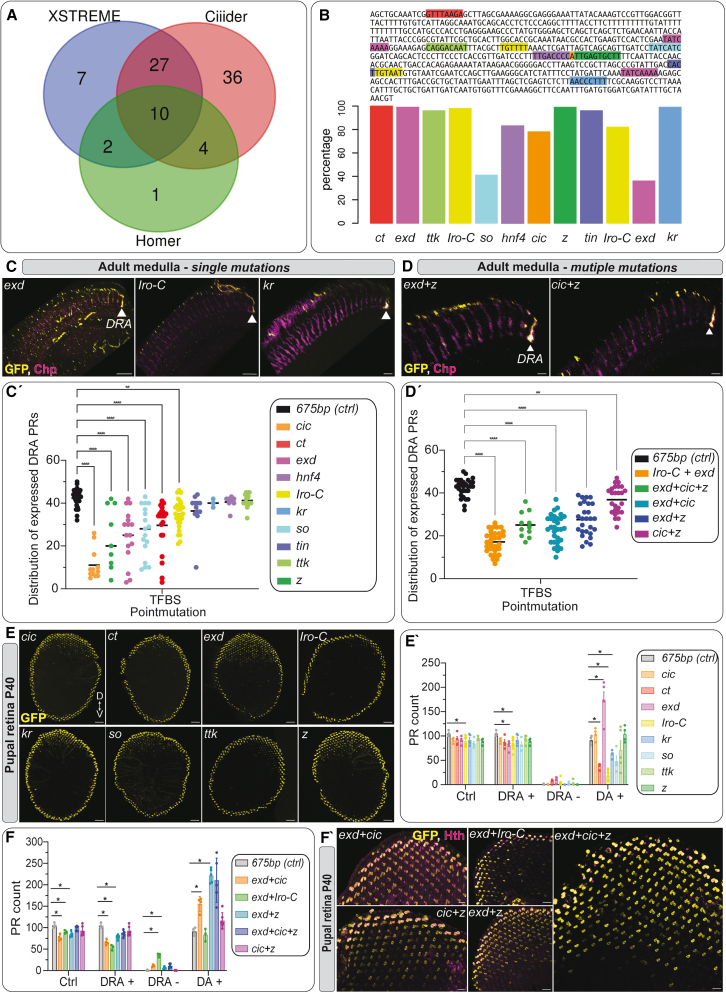
Identification and functional analysis of conserved TFBSs within the 675 bp minimal DRA enhancer (A) Venn diagram showing the overlap among three independent motif discovery tools (Ciiider,^32^ XSTREME,^33^ and HOMER^34^), revealing ten conserved TFBMs identified across all *Drosophilidae*. (B) Schematic representation of the 675 bp minimal enhancer indicating the position and identity of each TFBM (color-coded), with a bar graph below quantifying their conservation in percentage if TFBM is present in the alignment across analyzed *Drosophila* species. (C) Adult medulla expression patterns of new DRA enhancer reporter lines carrying single point mutations in conserved TFBSs. (C′) Quantification of DRA photoreceptor (PR) distribution within DRA columns for single TFBS mutants, showing high variability among individual mutations. (D) Corresponding adult medulla expression patterns of combined TFBS point mutant reporter lines. (D′) Quantification of DRA PR distribution for combined TFBS point mutations, revealing no complete loss of DRA PRs but marked variability between combinations. (E) P40 pupal retinas showing GFP reporter expression in R7 and R8 for single TFBS mutants. All variants retain labeling in rim PRs, while DA expression levels vary among mutants. (E′) Quantification relative to the 675 bp control shows *exd* and *Iro-C* TFBS mutants deviating most within the DRA. GFP-negative counts remain unchanged (DRA-), while the DA shows the greatest variability, largest in *exd*, smallest in *Iro-C*. (F) Quantification of combined TFBS mutations demonstrates variable effects on enhancer activity. Combinations including exd (except the triple combinations) show clear DRA-specific changes, while *exd+cic+z* results in the strongest dorsal expansion. (F′) Representative expression patterns for combined TFBS mutations. Statistical analyses: one-way ANOVA (C′ and D′) and unpaired *t* test with Welch correction (E′ and F); significance levels: *p* < 0.05 = ∗, *p* < 0.01 = ∗∗, *p* < 0.001 = ∗∗∗. Error bars: mean ± SEM. Sample sizes: *N* = 3 (*kr, exd+Iro-C* (C′ and F)); 4 (Ctrl, *cic, ct, so, z, exd+cic* (E′ and F)); 5 (*exd, ttk, exd+z, exd+cic+z* (E′ and F)); 6 (*cic+z* (F)); 7 (*tin, z* (E′)); 8 (*Iro-C* (E′)); 11 (*so* (C′)); 13 (Ctrl (D′), *exd, ttk* (C′)); 16 (*ct* (C′)); 25 (*cic+z* (D′)); 27 (*exd+z* (D′)); 30 (Ctrl (C′), *exd+Iro-C*, *exd+cic* (D′)). Error bars: mean ± SEM. Scale bars, 50 μm (E and F′) and 20 μm (C and D).

We next investigated the transcriptional regulation of the identified minimal 675 bp DRA enhancer within the *hth* locus, which was detected within the highly conserved *hth* locus of all 149 analyzed Drosophilidae species (see methods), suggesting a critical regulatory role maintained throughout Drosophilid evolution. Next, to identify potential transcription factor binding sites (TFBSs), we analyzed the 675 bp sequence for short regulatory motifs that are evolutionarily conserved across all 149 species. We aligned the orthologous enhancer sequences and analyzed them using three complementary motif discovery tools, Ciiider,^32^ XSTREME,^33^ and HOMER^34^ (Figure 3A). TFBM enrichment was evaluated against two background datasets (see methods), and predicted motifs were compared to position weight matrices from the FlyFactorySurvey database.^35^ By integrating results from these approaches, we identified ten potential, evolutionarily conserved TFBSs within the 675 bp enhancer (Figures 3A and 3B). These putative binding sites provided the basis for our subsequent *in vivo* analyses aimed at testing their functional contribution to enhancer activity.

To functionally assess the importance of these conserved motifs, we introduced individual, single-nucleotide substitutions into the core of each predicted binding site within the 675 bp enhancer, by changing a central purine to pyrimidine or vice versa (see methods). Each mutated enhancer variant was then cloned into the same reporter construct backbone used for the original 675 bp minimal Gal4 construct and tested for its ability to drive expression in DRA photoreceptors. Analysis of adult brains revealed that none of the single point mutations completely abolished DRA-specific reporter expression (Figures 3C and S4A). Instead, membrane-tagged UAS-GFP reporter expression persisted in DRA inner photoreceptors, although several mutants displayed expanded expression into VR photoreceptors and, particularly, point mutations in putative exd and hnf4 motifs, also exhibited ectopic expression in neurons of the protocerebrum (Figure S4B). Quantification of GFP-positive DRA columns revealed varying degrees of variability among different brains of the same single mutations (Figure 3C'). For instance, mutations in putative binding sites for the transcription factors Krueppel (*kr,* FBgn0001325), Hepatocyte nuclear factor 4 (*hnf4,* FBgn0004914), Tinman (*tin*, FBgn0004110), and Tramtrack (*ttk,* FBgn0003870) showed expression comparable to the control enhancer (∼40 DRA columns), whereas mutations in putative binding sites for the transcription factors Capicua (*cic,* FBgn0262582), Cut *(ct,* FBgn0004198*),* Exd *(exd,* FBgn0000611*),* Sine oculis *(so,* FBgn0003460) and Zeste (*z,* FBgn0004050) caused strong variability (range: 4 to 40 DRA columns). Mutation of Iro-C sites produced an intermediate phenotype (range: 20 to 40 columns).

Since none of these single mutations eliminated enhancer activity, we decided to test whether multiple TFBSs act cooperatively to control and refine DRA-specific Hth expression, in analogy to what has been shown in other systems.^15^ We therefore generated reporters carrying combinations of mutations, focusing on sites that showed the strongest single-mutant variability (*cic, exd,* and *z*) or are known for their biological relevance in DRA specification (*Iro-C*).^16^^,^^17^ Even when combining mutations, reporter expression in the adult medulla was never entirely lost (Figures 3D, D′, S4B and S4C). However, quantification revealed significantly fewer GFP-positive DRA photoreceptors in all combinatorial mutants, when compared to the control, with reduced variability between replicates compared to the single mutant constructs. The most pronounced reduction in DRA expression occurred in the *exd+Iro-C* combination, which decreased expression in DRA photoreceptors by approximately 50% (∼50 DRA PRs). Combinations involving exd also caused the expansion of reporter expression into the DA, particularly *exd+cic+z* and *exd+z* (Figure S4C' and C''). These results indicate that the DRA expression of the 675 bp minimal DRA enhancer indeed seems to be regulated by a network of TFBSs. While single mutations had no strong effect on enhancer function, combinatorial mutations suggest both redundant and additive interactions among motifs.

As some reporter expression changes in adults appeared subtle, particularly in posterior medulla regions, we also examined enhancer-mediated expression during the mid-pupation (P40), when DRA specification and synaptogenesis are occurring (Figures 3E and 3F′). All single-mutant variants manifested robust GFP reporter expression in rim R7 and R8 photoceptors, similar to the unmutated enhancer. Within the DRA, however, GFP levels varied among mutants: Expression was reduced in some (*ct, Iro-C, kr, so*, and *ttk*), whereas expanded into the DA, in others (*cic, exd,* and *z*, Figure 3E). This expansion into the DA was the most prominent phenotype, with up to 200 additional DA photoreceptors labeled in *exd, cic* or *z* single mutants, compared to roughly 75 additional DA cells in ct and *Iro-C* mutants (Figure 3E'). Importantly, no loss of reporter expression was observed in the DRA. But consistent with our adult data, combinations involving exd, except for the triple mutant (*exd+cic+z*), produced a loss of expression in the DRA (*exd+cic*: ∼75/85; *exd+Iro-C*: ∼50/95; *exd+z*: ∼80/95 DRA PRs show expression). In addition, *exd+z* and *exd+cic+z* combinations resulted in an extensive expansion of expression into the DA, labeling up to 250 non-DRA R7/R8 cells (Figure 3F and 3F′). Together, these findings support a model in which multiple TF inputs cooperatively regulate the expression of the DRA enhancer. Most mutations result in derepression, suggesting a substantial repressive contribution to refining enhancer expression. However, since a relatively small number of putative TFBS motifs was tested and loss of TF binding was not directly validated *in vitro*, the precise effect of individual sites, as well as their cooperative action, remains to be determined.

### The role of homothorax in the DRA is an evolutionary novelty specific to higher flies

Having defined the putative regulatory logic controlling *hth* expression in *Drosophila*, we next decided to test whether the transcription factor Hth plays a comparable role as a master regulator of DRA specification in more distantly related insect species whose eyes also possess a DRA. We chose three species with distinct visual ecologies: the honeybee (*Apis mellifera*, Hymenoptera), the monarch butterfly (*Danaus plexippus*, Lepidoptera), and the yellow fever mosquito (*Aedes aegypti*, Diptera, Figure 1C). These species belong to divergent lineages with well-characterized navigational behaviors, ranging from celestial-based foraging (honeybees)^36^ to long-distance migration (monarch butterflies)^37^ and host seeking (mosquitoes).^38^^,^^39^ As advanced genetic tools are still limited in these species, we generated polyclonal antibodies against each of their predicted Hth protein sequences in order to enable cross-species comparison of expression patterns during DRA development.

**Figure 4:**
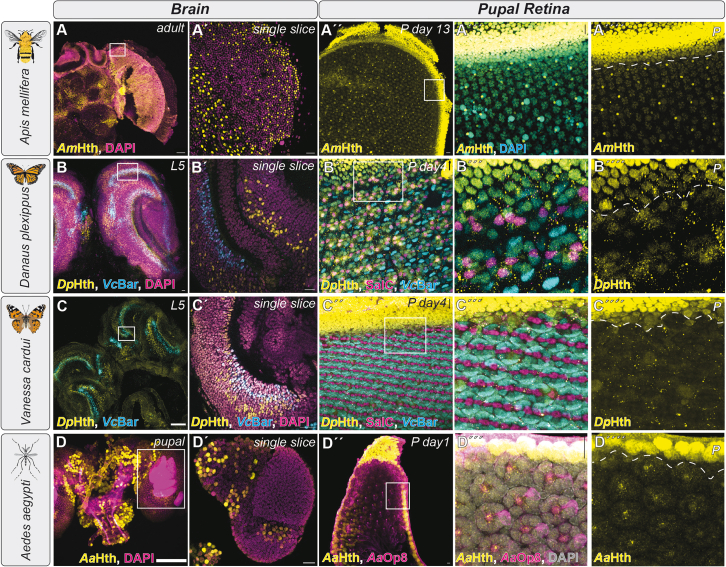
Absence of Homothorax from DRA ommatidia beyond higher diptera (A and A′) Homothorax *(*Hth, yellow) immunostaining in the adult brain of a honeybee worker (*Apis mellifera*) labels distinct neuronal population. (A'' and A''') Pupal retina (13-day-old worker) showing the most dorsal ommatidia. The boxed region marks the ROI shown at higher magnification. Hth is clearly localized to the pigment rim and bristle cells but absent from ommatidia adjacent to the rim. (B and B′) Anti-Hth immunostaining in the larval brain of the monarch butterfly (*Danaus plexippus*) labels specific cells distinct from Bar-positive (cyan) cell populations. (B'' and B'''') Dorsal pupal retina (4-day-old) labeled with Anti-Bar (outer photoreceptors) and Anti-Spalt *(Sal*; inner photoreceptors). Anti-Hth signal appears only as background autofluorescence, with no specific labeling. (C and C′) Whole-mounted fifth instar larval brain of *Vanessa cardui* stained with monarch Anti-Hth antibody (yellow) and Anti-Bar (cyan) antibody, confirming non-overlapping expression. The *Dp*Hth antibody cross-reacts and functions in *Vanessa cardui.* (C'' and C'''') Control staining in a 4-day-old *V. cardui* pupal retina shows strong *hth* expression in the pigment rim but not in adjacent ommatidia. (D and D′) Anti-Hth immunostaining in the pupal brain of *Aedes aegypti* labels distinct neuronal sets. (D'' and D'''') Pupal retina (1-day-old) labeled with *Aa*Op8 (marking all R7 photoreceptors except those in the dorsal region). Hth localization is confined to the pigment rim and pigment cells, with no detectable signal in photoreceptors. In all brain samples, DAPI (magenta) was used to label all nuclei. In all retinal close-up images, “P” denotes the pigment rim, with the dashed line outlining the area adjacent to the photoreceptors. Per species: *N* = 3 (brain) and *N* = 5 (retina). Scale bars, 50 μm (brain) and 20 μm (retina).

Antibodies were validated using dissected brain tissue from each taxon. Since *D. plexippus* eggs were collected from the wild in San Diego, California (USA), resulting in variable viability, we also included the closely related painted lady (*Vanessa cardui*), which was maintained under controlled laboratory conditions. In all four species, the newly generated Anti-Hth antibodies labeled distinct subsets of cells and nuclear signal was confirmed via co-labeling with DAPI (Figures 4A–4D′). Localizing the DRA regions in the developing retinas of these species proved challenging due to the limited availability of specific molecular markers during pupal stages. We therefore relied on previous studies to guide dissections. In butterflies, particularly *D. plexippus,* the DRA was described as a distinct patch-like region near the antennal base. In adult monarch retinas, this region was identified using a UV opsion antibody and UVRh mRNA *in situ* hybridization, confirming its molecular identity.^40^^,^^41^ However, our experiments were performed during pupal stages when TFs regulating photoreceptor cell fates are known to be expressed,^2^ and in which opsin transcripts are not yet detectable, precluding the use of these markers to positively label the DRA directly. Similarly, in *A. mellifera*, early anatomical descriptions of the adult retina also reported a rather localized patch-like arrangement of DRA ommatidia.^42^ In contrast, in *Aedes aegypti*,^43^ Opsin 9 is already restricted to the most dorsal pupal retina (the putative DRA) and Opsin 8 expressed in all other R7 cells (Figure 4D'' and 4D'''). Surprisingly, in all four species, *hth* expression was absent from all pupal photoreceptors (Figures 4A''–4D''). Instead, strong Anti-Hth labeling was consistently detected in the pigment rim right adjacent to the developing retina, as well as in bristle cells, hence a pattern resembling *Drosophila*.^18^ To ensure no retinal expression was overlooked, entire retinas were mounted and systematically scanned by confocal microscopy, and multiple ROIs were analyzed, with particular emphasis on the dorsal periphery. When tested on *Drosophila* pupal retinal tissue, the *Aedes aegypti* Anti-Hth antibody cross-reacted and labeled DRA R7 and R8 photoreceptors, whereas *Apis* and *Danaus* antibodies did not (Figure S1). Taken together, these results demonstrate that, while *hth* expression in the pigment rim is conserved across insect lineages, its presence in developing DRA photoreceptors is not. The role of the transcription factor Hth in specifying DRA identity might therefore represent a derived feature of Neodipteran evolution.

**Figure 5:**
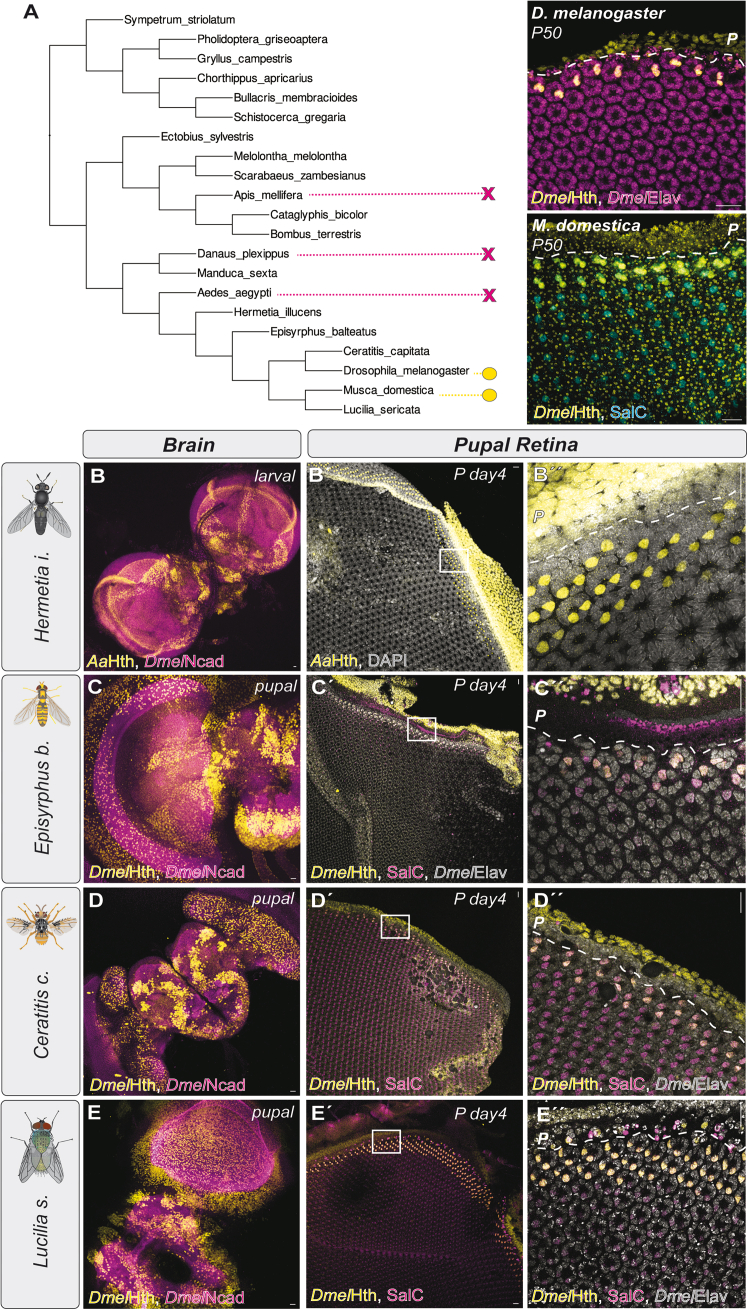
Conservation of *hth* expression across higher diptera (A) Phylogenetic depiction shows *hth* expression in the DRA photoreceptors of *Drosophila melanogaster* and *Musca domestica* (highlighted with a yellow circle and stainings)*,* while absent in more basal insects such as mosquitoes (*Aedes aegypti*), as well as in non-dipteran species, including butterflies (*Danaus plexippus*) and honeybees *(Apis mellifera)* (indicated by magenta x). Pupal retina of *Musca* shows strong Anti-Hth labeling in inner DRA photoreceptors (R7 and R8, indicated by Anti-Sal in cyan) and the pigment rim, organized in a narrow band comparable to *Drosophila*. (B–E) Pupal retinas of selected neodipteran species representing different evolutionary clades between *Drosophila* and *Aedes*. All samples were staged to mid-metamorphosis, when retinas are non-pigmented. *Sal* labels inner photoreceptors (magenta), Anti-Elav marks all photoreceptors (gray), and Anti-Hth staining is shown in yellow. In all tested species, *hth* is expressed in a narrow dorsal band of ommatidia corresponding to the DRA, as well as in the pigment rim (“P”). *N* = 5 per species. Scale bars, 50 μm (B–E) and 20 μm (B′–E'').

To further test this idea, we next asked when the *hth* expression in DRA photoreceptors first emerged during dipteran evolution. Since the Hth protein was absent even from the DRA of a basal Dipteran species such as *Aedes*, we aimed to determine whether this *hth* expression is common to all higher flies. As previously reported,^44^ an Anti-Hth antibody raised against the *Drosophila* Hth protein labeled both inner photoreceptors (R7 and R8) in *Musca domestica* (housefly), clearly marking a narrow band of dorsal ommatidia, as well as the pigment rim, thereby closely matching the pattern observed in *Drosophila* (Figure 5A). We next examined four additional diurnal dipterans representing major higher fly superfamilies with known DRAs^45^^,^^46^^,^^47^: *Hermetia illucens* (soldier fly, Stratiomyomorpha), *Episyrphus balteatus* (hoverfly, Empidoidea), *Ceratitis capitata* (Mediterranean fruit fly, Tephritoidea), and *Lucilia sericata* (blowfly, Oestroidea). Criteria for selection were the availability of developing stages (for precise developmental timing), and they had to be commercially accessible. Larval and pupal brain tissue, for the validation of antibody cross reaction (Figures 5B–5E) and mid-pupal stage retinas (prior to pigmentation) were analyzed using cross-reacting Anti-Hth in combination with antibodies against the transcription factor Embryonic lethal abnormal vision (*Elav*, FBgn0260400, all photoreceptors) and Spalt (inner photoreceptors). In each higher fly species examined, Hth protein was consistently and clearly localized in a narrow dorsal band of dorsal marginal photoreceptors and strongly present in the pigment rim (Figures 5B'–5E′). Species-specific differences only affected the width of the DRA region, consistent with differences in compound eye size across these species.

In summary, *hth* expression in DRA photoreceptors is conserved across higher flies, whereas it is absent in more basal dipterans such as mosquitoes, as well as in other insect orders. The recruitment of *hth* into the molecular DRA specification program, therefore, appears to represent a Neodipteran innovation that coincided with the diversification of higher diptera.

### Putative molecular changes at the evolutionary transition from lower to higher flies

**Figure 6:**
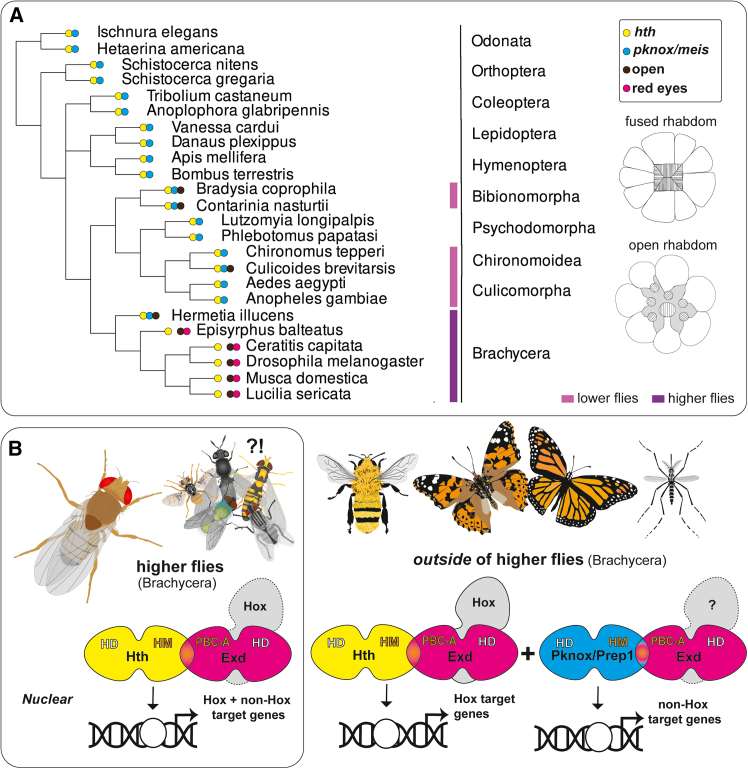
Comparison of *pknox* and *homothorax* across insects (A) Phylogenetic tree illustrates that ancestral insects possess both *hth* and *pknox/meis*. In higher diptera, *pknox* is lost with the evolution of superposition eyes (indicated by “open”) and red eye pigmentation, while Hth is retained as the primary DRA regulator. Non-Neodipteran insects putatively use *pknox* in their DRA. *Hermetia illucens* expresses both, with Hth protein present in DRA photoreceptors as shown in Figure 5B'. (B) Schematic representation of the hypothesized regulatory mechanisms governing DRA photoreceptor identity in different insect species. In higher diptera, *hth* is expressed in inner DRA photoreceptors. In *Drosophila*, the Hth protein is known to interact with Exd via its HM domain and Exd's PBC-A domain, facilitating Exd nuclear localization.^23^ This complex, potentially together with Hox proteins, regulates both Hox-dependent and non-Hox target genes. In contrast, basal insect genomes exhibit both *hth* and *pknox (prep1)*. While Hth likely retains its role in activating Hox target gene in trimeric complex with Exd/Hox, Pknox possibly forms a similar complex with Exd^49^ to regulate non-Hox target genes, putatively including those involved in specifying DRA identity.

Since Hth does not appear to be the universal regulator of DRA photoreceptor development, we considered different scenarios, distinguishing between convergent evolution of DRAs vs. a putatively conserved, molecular mechanism. On one hand, an unknown transcription factor, binding to different binding sites, might fulfill this role outside of higher flies. On the other hand, we reasoned that any factor capable of replacing Hth could play this role outside of higher flies, thereby using the same, potentially conserved binding motifs. Previous work by^16^ showed that Hth and Exd, a TALE-class transcription factor,^21^^,^^24^^,^^48^ act together in specifying the DRA. Through its Homothorax-Meis (HM) domain, Hth binds to Exd's PBC-A domain,^23^ enabling Exd's nuclear import and subsequent activation of DRA-specification. This process is strictly Hth-dependent in *Drosophila*, since Hth is the only *Drosophila* gene containing an HM domain.^21^ We therefore reasoned that in insects where Hth protein is absent from developing DRA photoreceptors, such as honeybees, butterflies, and mosquitoes, another HM-domain transcription factor may substitute for it. In such a scenario, DRA photoreceptor development would still be Exd-dependent, yet independent of Hth. Using TBLASTN searches across insect genomes (NCBI), we indeed identified Pknox (also known as Prep1 in vertebrates), sharing sequence similarity with the Hth protein, including the conserved HM domain required for Exd binding. Our phylogenetic analysis confirmed the presence of *pknox* orthologs in the genomes of all available, non-neodipteran insects and confirmed their absence from the *Drosophila* genome (Figure 6A). Importantly, the human Prep1 protein has been shown to interact with Exd and induce nuclear localization when transgenically expressed in *Drosophila,* suggesting an evolutionarily conserved functional equivalence between Pknox and Hth when interacting with Exd.^49^ Hence, Hth and Pknox proteins therefore seemed to act as alternative co-factors for Exd outside of neodipteran insects, regulating both Hox targets and non-Hox targets, and Hth assumed both roles in higher Diptera, due to the loss of the *pknox gene*. Further phylogenetic analyses revealed that the loss of *pknox* and emergence of *hth* expression in the DRA indeed occurred within a similar evolutionary time window, at the transition from lower to higher flies (Figure 6A). Interestingly, this time window coincides with the evolution of open rhabdoms in higher flies, which changed photoreceptor optics quite drastically.^5^ Simultaneously, photoreceptor photochemistry also changed through the emergence of red-pigmented eyes expressing the newly evolved Rhodopsin1 (*ninaE,* FBgn0002940), whose metarhodopsin absorbs longer wavelength light, thereby enabling highly efficient isomerization (Figure 6A).^50^

Together, the correlations unearthed via these phylogenetic analyses raise the possibility that both changes in photoreceptor function and transcriptional regulation of the DRA fate could have co-evolved during the emergence of higher diptera, providing an alternative model for how Hth became the principal regulator of DRA specification in higher fly lineages (Figure 6B).

## Discussion

The DRA is a nearly ubiquitous retinal specialization found in the dorsal periphery of virtually all insect eyes, raising the question of whether DRAs are homologous structures derived from a common ancestor or arose independently through convergent evolution. To approach this, we investigated the regulatory logic of *hth* expression in the *Drosophila* DRA and *hth* expression in developing DRA photoreceptors across insect species.

### Regulation of DRA-specific *hth* expression

Enhancer specificity provides a central mechanism by which TFs achieve precise spatial and temporal control of gene expression.^51^ These regulatory elements integrate multiple TF inputs through clustered binding sites, functioning as logic gates that determine cell-type-specific transcriptional outcome.^52^ In this study, we identified a minimal 675 bp enhancer sufficient to drive *hth* expression specifically in both developing and adult DRA photoreceptors R7 and R8 of *Drosophila*. In addition, reporter assays revealed additional expression of the minimal enhancer in R7 and R8 along the VR, as well as - less frequently - in the DA. Interestingly, this “leaky” expression is most apparent during pupal stages, when DRA specification occurs^18^ and becomes less prominent in the adult. This indicates that, while its small size makes it amenable to site-directed mutagenesis, the minimal enhancer lacks a repressive component involved in restricting expression to the DRA. Nevertheless, CRISPR-mediated deletion of the enhancer produced viable flies lacking a functional DRA, without the ability to detect polarized light, but with an otherwise normally developed visual system. Furthermore, homozygous enhancer deletion did not abolish *hth* expression in the pigment rim, indicating the existence of a separate enhancer controlling this domain, whereas the 675 bp element is both necessary and sufficient for the DRA-specific expression of *hth*. Deletion of this small *cis*-regulatory sequence, therefore, leads to the specific elimination of an entire retinal detector array and the orientation behavior it guides, which exists across insects. Interestingly, very few insect species have so far been described as lacking a retinal DRA region for the detection of linearly polarized skylight.^8^ Here we show that the molecular removal of an entire DRA is possible, at least in *Drosophila*, and the presence or absence of similar enhancer sequences might serve to specify similar structures across insects.

Comparison of the minimal enhancer across Drosophilidae identified at least ten putative, evolutionarily conserved TFBSs as candidate regulatory inputs for regulating DRA specification. Site-directed mutagenesis of all motifs individually did not abolish enhancer activity, and most perturbations resulted in quantitative rather than qualitative changes in expression. These findings are consistent with the well-understood concept of combinatorial regulation. However, only single-nucleotide substitutions were introduced per TFBS, and disruption of TF binding was not validated *in vitro*. Furthermore, the predominance of derepression phenotypes and the absence of clearly identifiable strong activator sites suggest that additional regulatory inputs may not yet have been captured within the analyzed motifs. Several mutations led to an increased expression in the DA, resulting in an expression domain reminiscent of the ombQuadroon phenotype, a gain-of-function allele of the T-box transcription factor Omb.^14^^,^^16^^,^^53^ Omb, together with Iro-C transcription factors, acts downstream of *wg* to induce *hth* expression in the dorsal rim. Consistent with this finding, mutating Iro-C binding sites within the minimal enhancer caused a decrease in “leaky” DA expression and, in a combinatorial context, contributed to partial loss of DRA expression. These results are compatible with a model in which the enhancer integrates both activating and repressive inputs from the *wg* pathway effectors and dorsal selector genes. However, given the modest phenotypic effects of individually mutated motifs, further experiments, such as larger deletions, motif cluster disruption, or direct binding assays, might be required for a more detailed definition of the minimal enhancer’s regulatory logic.

### Evolution of hth in DRA specification

Specialized DRAs are found across nearly all insect lineages. The *Drosophila* retina, with its well-characterized mosaic organization,^53^^,^^54^ serves as a powerful model system for studying how combinatorial transcriptional codes refine photoreceptor identity.^55^ Transcription factors such as Salm, Pros, Bar, Dve, Ss, and Hth were identified to play important roles in specifying different photoreceptor subtypes, and ultimately rhodopsin choice.^10^^,^^18^^,^^56^^,^^57^^,^^58^^,^^59^^,^^60^^,^^61^^,^^62^ Recent studies have shown that the expression of most of these factors is deeply conserved across insect species (*salm, pros, Bar, dve, ss*, 2, 11). Interestingly, similar insight so far has remained missing for the specification of DRA photoreceptors. In *Drosophila*, *hth* is both necessary and sufficient to induce DRA fate during pupal development.^18^ One could have therefore expected the transcription factor Hth to be a universal DRA regulator of DRA fate across species, yet the Hth protein is missing from the DRA of all insect species tested, except for higher flies. Besides convergent evolution, our phylogenetic analysis provides an alternative molecular scenario for DRA specification being evolutionarily conserved despite Hth being absent from non-Neodipteran retinas. Pknox/Prep1 is a second TALE-class transcription factor sharing structural homology with Hth, including a conserved HM domain.^20^^,^^23^^,^^49^ The gene encoding Pknox exists in all non-Neodipteran insect genomes examined, the Pknox protein interacts with *Drosophila* Exd and promotes its nuclear localization in *Drosophila*,^49^ highlighting its functional conservation. Importantly, this scenario does not follow the classical model of gene duplication followed by neofunctionalization. Instead, it suggests an evolutionary trajectory in which an ancestral TALE-class transcription factor (likely Pknox) could have mediated Exd-dependent DRA specification, and only in higher Diptera *hth* was recruited to assume this role while *pknox* was lost. Such a shift is reminiscent of regulatory reassignment rather than straightforward neofunctionalization. Distinguishing between convergent evolution of DRA specification versus this hypothesis will require a deeper understanding of *pknox* expression and function in non-Neopdipteran insects. In particular, approaches such as ATAC-seq to identify accessible regulatory regions, combined with genome editing strategies to mutate or delete *pknox* regulatory sequences, would help clarify whether Pknox indeed fulfills a role analogous to Hth in DRA specification.

Interestingly, the first appearance of Hth protein in DRA photoreceptors appears to coincide with major optical and structural innovations in the dipteran eye. The emergence of neural superposition eyes in higher flies, characterized by open rhabdomeres and enhanced visual sensitivity, marks a key evolutionary shift from the fused rhabdom of basal insects. Neural superposition allows photoreceptors viewing the same visual axis to converge onto a single lamina cartridge, optimizing both sensitivity and spatial resolution.^5^ This innovation likely evolved once within Diptera and required molecular factors such as Eyes shut/Spacemaker (*Eys,* FBgn0031414), which promotes rhabdomere separation.^63^ In parallel, higher Diptera developed red screening pigments that transmit long-wavelength light, contrasting with the dark pigments of basal insects.^50^ Optical and structural transitions, including open rhabdom organization, altered spectral filtering, and red-shifted rhodopsins, coincide with the recruitment of the transcription factor Hth as the DRA regulator in higher diptera. Instead of reflecting a straightforward regulatory substitution, the shift we describe here may be an example of Developmental System Drift (DSD),^64^ whereby the molecular components of a regulatory network are modified over evolutionary time while the resulting morphology and function of the DRA remain preserved. In this context, the TALE-exd module is retained as the core regulatory logic, whereas the specific TALE factor deployed (Pknox versus Hth) differs between lineages. We propose that such regulatory changes might illustrate how conserved sensory structures can be maintained through evolutionary flexibility at the level of gene regulatory networks, balancing structural conservation with molecular innovation.

### Limitations of the study

Several limitations of this study can be considered. Our experimental work primarily focused on the role of Homothorax (*hth*) in flies and beyond, while any potential role of Pknox/Prep1 in DRA specification was proposed solely based on correlations revealed by our phylogenetic analyses (Figure 6A). In the future, a direct investigation of *pknox* expression and function in non-Brachyceran species will be highly informative, yet requiring species-specific probes for *in situ* hybridization experiments, or suitable, cross-reactive antibodies. Soldier flies may represent a particularly promising model system in this context, as they appear to combine features of both regulatory programs, expressing *hth* in DRA photoreceptors, while retaining the *pknox* gene at the genomic level. Additionally, future studies should investigate how differences in photoreceptor optics and changes in overall DRA architecture necessitate each other. Finally, while a number of site-directed TFBS were analyzed via mutagenesis, identifying apparent derepressive roles for some of them, the full combinatorial regulatory logic of the minimal *hth* enhancer remains only partially resolved. Further work using CRISPR-based mutagenesis could be used for an even larger-scale, systematic dissection of transcription factor interactions and help define the minimal regulatory code underlying DRA specification in *Drosophila*.

## Resource availability

### Lead contact

All requests for additional information and resources should be directed to the lead contact, Mathias F. Wernet (mathias.wernet@fu-berlin.de).

### Materials availability

All unique reagents and fly strains generated in this study are available from the lead contact upon request.

### Data and code availability

• All data reported in this paper will be shared by the lead contact upon request.
• This study did not generate any new codes.
• Any additional information required to reanalyze the data reported in this paper will be shared by the lead contact upon request.

## Acknowledgments

The authors would like to thank C. Desplan, E. Wimmer, J. Aldrich, O. Akbari, and R. Menzel for generously sharing crucial reagents, as well as G. Belušič and two anonymous reviewers for their conceptual feedback. This work was supported by the 10.13039/501100001659Deutsche Forschungsgemeinschaft (DFG) through the grant SPP 2205 (K.N. and M.F.W), and 10.13039/100000181AFOSR grant FA 8655-23-1-7049 (M.F.W.).

## Author contributions

Conceptualization, H.R., M.S., M.W.P., K.N., and M.F.W.; data curation, H.R. and M.S.; formal analysis, H.R., M.S., A.S., and J.B.; funding acquisition, K.N. and M.F.W.; investigation, H.R., M.S., A.S., and J.B.; methodology, H.R., M.S., M.W.P., K.N., and M.F.W.; project administration, H.R. and M.S.; supervision, K.N., M.W.P., and M.F.W.; resources, K.N., M.W.P., and M.F.W.; visualization, H.R. and M.S.; validation, H.R. and writing – original draft, H.R.; writing – review and editing, H.R., M.S., M.W.P., K.N., and M.F.W.

## Declaration of interests

The authors declare no competing interest.

## Declaration of generative AI and AI-assisted technologies in the writing process

The authors did not use AI tools in the writing and conduct of this study.

## STAR★Methods

### Key resources table

**Table:** 

REAGENT or RESOURCE	SOURCE	IDENTIFIER
**Antibodies**		

Guinea pig Anti-*Am*Hth	This study	N/A
Rabbit Anti-*Aa*Hth	This study	N/A
Rat Anti-*Aa*Op1	Perry Lab (unpublished)	N/A
Guinea pig Anti-*Aa*Op8	Perry Lab (unpublished)	N/A
Rat Anti-Cadherin	DSHB	Cat#DN-Ex#8; RRID: AB_528121
Mouse Anti-Chaoptin	DSHB	Cat#24B10; RRID: AB_528161
Guinea pig Anti-*Dmel*Hth	Gift from C. Desplan	N/A
Guinea pig Anti-*Dp*Hth	This study	N/A
Mouse Anti-Elav	DSHB	Cat#Elav-9F8A9; RRID: AB_528217
Rabbit Anti-GFP pAB	Thermo Fisher Scientific	Cat#A-11122; RRID: AB_221569
Rabbit Anti-SalC	Perry Lab (unpublished)	N/A
Mouse Anti-Rh3	Gift from J. Aldrich	N/A
Rabbit Anti-Rh6	Gift from M.W. Perry	N/A
Rat Anti-*Vc*Bar	Gao et al.^65^	N/A
Donkey Anti Rabbit Alexa Fluor 488	Jackson ImmunoResearch Lab	Cat# 711-545-152; RRID: AB_2313584
Donkey Anti Guinea pig Alexa Fluor 488	Jackson ImmunoResearch Lab	Cat#706-545-148; RRID: AB_2340472
Donkey Anti-Rat Cy3	Jackson ImmunoResearch Lab	Cat#712-165-153; RRID: AB_2340667
Donkey Anti Rabbit Cy3	Jackson ImmunoResearch Lab	Cat#711-165-152; RRID: AB_2307443
Donkey Anti-Mouse Alexa Fluor 594	Jackson ImmunoResearch Lab	Cat# 715-585-151; RRID: AB_2340855
Donkey Anti Mouse Cy5	Jackson ImmunoResearch Lab	Cat#715-175-151; RRID: AB_2340820
Donkey Anti Rabbit Cy5	Jackson ImmunoResearch Lab	Cat#711-175-152; RRID: AB_2340607
Donkey Anti-Rat Cy5	Jackson ImmunoResearch Lab	Cat#712-175-153; RRID: AB_2340672
Donkey Anti Guinea pig Cy5	Jackson ImmunoResearch Lab	Cat#706-175-148; RRID: AB_2340462

**Chemicals, peptides, and recombinant proteins**		

Normal Goat Serum	Jackson ImmunoResearch Lab	Cat# 005-000-01; RRID: AB_2336983
Normal Donkey Serum	Jackson ImmunoResearch Lab	Cat# 017-000-12; RRID: AB_2337258
DAPI	Sigma-Aldrich	Cat #28718-90-3
Quick-Load® Purple 100 bp DNA Ladder	NEB	N00551S
Quick-Load® Purple 1 kb Plus DNA Ladder	NEB	N0550SL
Agarose	Sigma-Aldrich	A9539
Ampicillin	VWR	N/A
rCutSmart^TM^ Buffer	NEB	B6004S
Subcloning Efficiency^TM^ DH5α^TM^ chemically competent cells	Invitrogen^TM^	18265017
EcoRI-HF	NEB	R3101S
Ethidium Bromid solution	Invitrogen^TM^	15585011
KpnI-HF	New England Biolabs	R3142S
LB Agar (Lennox)	Carl Roth	X965.1
LB Broth (Lennox)	Carl Roth	X964.1
NaAz (1% sodium azide)	Sigma-Aldrich	N/A
PFA	Sigma-Aldrich	158127
PBS pH7.4	Gibco^TM^	10010023
SapphireAMP® Fast PCR Master Mix	TaKaRa	RR350A
Schneider's Medium	ThermoFisher Scientific	R69007
SlowFade^TM^	ThermoFisher Scientific	S36936
SphI-HF	New England Biolabs	R31182S
T4 DNA Ligase	ThermoFisher Scientific	EL0011
TAE Buffer (50x)	ThermoFisher Scientific	B49
Triton^TM^ X-100	Sigma Aldrich	X100
UltraPure^TM^ Distilled Water	Invitrogen^TM^	10977015
VECTORSHIELD	Vector Laboratories	H-1000
XbaI	New England Biolabs	R0145S

**Critical commercial assays**		

PureLink^TM^ Quick Plasmid Maxiprep Kit	Invitrogen^TM^	K210006
PureLink^TM^ Quick Plasmid Miniprep Kit	Invitrogen^TM^	K210010
NucleoSpin Gel and PCR Clean-up Kit	Macherey-Nagel	740609.50
NanoDrop® 2000 spectrophotometer	Thermo Fisher Scientific	N/A

**Experimental models: Organisms/strains**		

*Aedes aegypti*	Gift from O. Akbari	N/A
*Apis mellifera*	Gift from R. Menzel	N/A
*Ceratitis capitata*	Gift from E. Wimmer	N/A
*Danaus plexippus*	This study	N/A
*Drosophila*: Canton S	Bloomington Drosophila Stock Center	RRID: BDSC_64349
*Drosophila*: cic Gal4	This study	N/A
*Drosophila*: cic+exd+z Gal4	This study	N/A
*Drosophila*: cic+exd Gal4	This study	N/A
*Drosophila*: cic+z Gal4	This study	N/A
*Drosophila*: ct Gal4	This study	N/A
*Drosophila*: 5P DRA Gal4 (1.5 kb)	This study	N/A
*Drosophila*: Mid DRA Gal4 (1.5 kb)	This study	N/A
*Drosophila*: 3P DRA Gal4 (1.5 kb)	This study	N/A
*Drosophila*: 675bp DRA Gal4	This study	N/A
*Drosophila*: exd Gal4	This study	N/A
*Drosophila*: exd+IRO-C Gal4	This study	N/A
*Drosophila*: exd+z Gal4	This study	N/A
*Drosophila*: hnf4 Gal4	This study	N/A
*Drosophila*: hth full-length DRA (GMR45D01) Gal4	This study reinserted	N/A
*Drosophila*: *hthΔ675bp*	This study	N/A
*Drosophila*: Iro-C Gal4	This study	N/A
*Drosophila*: kr Gal4	This study	N/A
*Drosophila*: Oregon R (isogenized)	Bloomington Drosophila Stock Center	RRID: BDSC_5
*Drosophila*: rh6 Gal4	Bloomington Drosophila Stock Center	RRID: BDSC_7459
*Drosophila*: so Gal4	This study	N/A
*Drosophila*: tin Gal4	This study	N/A
*Drosophila*: ttk Gal4	This study	N/A
*Drosophila*: UAS-mCD8:GFP	Bloomington Drosophila Stock Center	RRID: BDSC_5137
*Drosophila*: UAS-Hth	Wernet Lab	N/A
*Drosophila*: z Gal4	This study	N/A
*Episyrphus balteatus*	Katz Biotech AG	RRID: Schwebfliege
*Hermetia illucens*	md terraristik	N/A
*Lucilia sericata*	Manditen Zimmer	RRID: Gold-Fliegen
*Musca domestica*	Gift from Perry Lab	N/A
*Vanessa cardui*	Gift from Perry Lab	N/A

**Software and algorithms**		

Adobe illustrator (Ai) (v29.1)	Abode	N/A
Ciiider (v0.9)	Gearing et al.^32^	N/A
Fiji (v.2.14.0/1.54f)	Schindelin et al.^66^	https://fiji.sc
FlyFactorSurvey	Zhu et al.^35^	https://pgfe.umassmed.edu/ffs/
GraphPad Prism (10.2.2 (397))	Graph Pad Software	N/A
HOMER (v4.11)	Heinz et al.^34^	N/A
IMARIS (v.9.1.2)	Bitplane	N/A
MAFFT (v7.490)	Katoh et al.^67^	https://mafft.cbrc.jp/alignment/software/
MATLAB	MathWorks	N/A
NCBI	Sayers et al.^68^	https://www.ncbi.nlm.nih.gov/
NEBioCalculator	NEB	https://nebiocalculator.neb.com/#!/ligation
RStudio (v.2024.04.2 + 746)	N/A	https://www.rstudio.com/
R (v.4.4.1)	R Core Team	https://www.r-project.org/
SnapGene	N/A	https://www.snapgene.com
XSTREME (v4.11.2)	Grant et al.^33^	N/A

### Experimental model and study participant details

#### Animal husbandry

All insect species were maintained under controlled laboratory conditions unless otherwise noted. *Aedes aegypti*, *Danaus Plexippus, Musca domestica* and *Vanessa cardui* were reared at UC San Diego (CA, USA), while all other species were maintained at Freie Universität Berlin (Berlin, Germany). A full list of insect species and strains is provided in key resources table.

*Drosophila melanogaster* strains were raised on standard molasses-corn food at 25°C under a 12h light/dark cycle. *Aedes aegypti* larvae were reared at 27°C in water tanks with fish food and pupae were dissected at mixed stages due their short (∼2 days) pupal phase. *Apis mellifera* worker pupae were obtained from sealed brood combs and 7-day-old pupae with non-pigmented eyes were dissected. *Ceratitis capitata* larvae were raised on carrot agar, pupated in sand, and maintained at 25°C until dissection. *Danaus plexippus* were collected as eggs from milkweed in San Diego, reared at 26°C, and dissected 4 days after pupation. *Episyrphus balteatus* pupae were kept at room temperature and dissections were performed on 3-4-day-old pupae with unpigmented retinae. *Hermetia illucens* larvae were raised at 20°C in darkness and pupae were staged for 10 days prior to dissection. *Lucilia sericata* larvae were reared at 25°C in darkness and retinae were dissected on a day 4 of the 8-day pupal period. *Musca domestica* freshly pupated animals were collected and maintained 50 h at 25°C (12h light/dark) before dissection. *Vanessa cardui* caterpillars were reared individually on artificial diet at 25°C (12h ligh/dark) and pupae were dissected 4–5 days after pupation.

### Method details

#### Generation of transgenic fly lines

Target sequences were cloned into the pJaBa2-Gal4 vector backbone (AmpR, Gal4, mini-white, ori)^69^ using standard molecular techniques. Inserts were PCR-amplified, digested (KpnI, XbaI), gel-purified, ligated, and transformed into *E. coli* DH5α. Positive clones were identified by colony PCR, and plasmids were purified and verified by Sanger sequencing (Microsynth SEQLAB). For transgenesis, 35 μg of plasmid DNA was injected into *Drosophila* embryos for site-specific integration at the attP2 landing site (68A4, 3L) using a commercial injection service (Rainbow Transgenic Flies, Inc.).

Point-mutated variants were generated by exchanging single purine or pyrimidine nucleotides within the predicted binding motifs. When multiple identical binding sites were present in the same construct, all sites were mutated simultaneously, and no separation of individual sites was performed. Minimal enhancer constructs and point-mutated variants were synthesized by GenScript (Piscataway, NJ), cloned via XbaI/KpnI, and subcloned for functional analysis.

#### CRISPR-mediated mutagenesis

CRISPR/Cas9 mutagenesis of *hth/CG17117* locus was carried out by WellGenetic Inc. Upstream and downstream gRNAs were cloned under U6 promoters, and a donor plasmid was constructed in a pUC57-Kan backbone containing ∼1 kb homology arms flanking 3xP3-DsRed selection cassette with PiggyBac repeats. Embryos of w^1118^ were co-injected with gRNA plasmids, hs-Cas9, and donor DNA. DsRed-positive F1 progeny were screened and validated by PCR and sequencing, confirming a 675 bp deletion (Chr3R: 10578025–10578699) replaced by the PbacDsRed cassette.

#### Antibody production

New polyclonal antibodies were generated by GenScript (Piscataway, NJ) and purified to >80% purity.

Protein sequences used for antibody production were.

##### *Am*Hth

MAQPRYDDGGLHSSYLEGGGGGGGAGLYDPHGSARGVPGLHHSPHLGGMGPAHPQYPPPPPQPPQHVLAAAAAAAASAVPDVHKRDKDAIYGHPLFPLLALIFEKCELATCTPREPGVAGGDVCSSESFNEDIAVFSKQIRQEKPYYIADPEVDSLMVQAIQVLRFHLLELEKVHELCDNFCHRYISCLKGKMPIDLVIDDRESSKPPEMGNGLDGGGPRSTADSTSHTDGASTPDVRPPSSSLSYPGAGGNEDARSPGSGGTPGPLSAQAPASLDSSDPGKWCPRREWSSPPDAQRAASDARRGVLYSSVFLGSPGDASNASIGSGEGTGEEDDDSSGKKNQKKRGIFPKVATNILRAWLFQHLTHPYPSEDQKKQLAQDTGLTILQVNNWFINARRRIVQPMIDQSNRAVFPPLSAGPSGAYSPDPTMGYMMDGQAASMMHRPPGDPTFHNQYHYPEYYGHHL.

##### *Dp*Hth

MAQPRYDESLHGGGYMEGGAMYHEHRLSHPHIPPVHYPPPAAPAHALPGEPLVHKRDKDAIYGHPLFPLLALIFEKCELATCTPRDPGVAGGDVCSSESFNEDIAVFSKQIRQEKPYYIADPEVDSLMVQAIQVLRFHLLELEKVHELCDNFCHRYISCLKGKMPIDLVIDERETRPPDNGERSAPDSSHDGASTPDVRPPSSSLSYGGAVNDDVRSPGSGGTPGPLSQPPPQTLDPDADAMGKWCGSRREWSSPPDVARRVYSSVFLGSPGEYPGDASNASIGSGEGTGEEDDDTNGKKNQKKRGIFPKVATNILRAWLFQHLTHPYPSEDQKKQLAQDTGLTILQVNNWFINARRRIVQPMIDQSNRAVFPHAGPSGAYSPEATMGYMMDGQQMMHRPPADPAFHQGYAHYPAEYYGHHL.

##### *Aa*Hth

MAQPRYDDGLHGYGMDGGAAAMYDPHAGHRPPGLSGLPPHHSPHMNHAAAASVGMHGYHGTSSHVSPATSHMGAVQPDVHKRDKEAIYGHPLFPLLALIFEKCELATCTPREPGVAGGDVCSSDSFSEDVAVFSKQIRQEKPYYVADPEVDSLMVQAIQVLRFHLLELEKVHELCDNFCHRYISCLKGKMPIDLVIDERDTTKPPELGGANGEGRSNADSTSHTDGASTPDVRPPSSSISYSGAVNDDVRSPGSGSTPGPLSAQPPPGLDTPDPDGRWCSRRDWSSPPDSRVDANRRVLYSSVFLGSPVAVDFTSTSIQGDASNTSIGSGEGTGEEDDDSNGKKNQKKRGIFPKVATNILRAWLFQHLTHPYPSEDQKKQLAQDTGLTILQVNNWFINARRRIVQPMIDQSNRAVYTHPGPSAGYPDAMSYMMDGQAQMMHRPPGDPTFHQGYHYPPAEYYPHHL

#### Immunohistochemistry and imaging

Larval discs, pupal, and adult *Drosophila melanogaster* retinas were dissected in ice-cold Schneider's Insect Medium (ThermoFisher Scientific, #R69007) following.^70^ Tissues were fixed in 4% PFA in 1xPBS for 15 min (adult) or 20 min (larval and pupal). Larval discs and pupal retinas remained attached to the brain until the final step of the staining.

For non-*Drosophila* species, pupal retinas were dissected in ice-cold PBS (Gibco™, #10010023) and fixed for 15 min in 4% PFA. After fixation, tissues were rinsed in PBS and washed in PBS-T (PBS +0.4% Triton X-100 (Sigma Aldrich, #X100) for *Drosophila*, 0.2% for other insects). Samples were blocked for 30 min in 5% Normal Donkey or Goat Serum (Jackson ImmunoResearch Labs) in PBS-T, then incubated overnight at 4°C with primary antibodies diluted in the same blocking solution. Following three 20-min washes in PBS-T, samples were incubated overnight in secondary antibody solution (1:500; protected from light). After final washes, tissues were stained with DAPI (1x; Sigma-Aldrich, #28718-90-3) and mounted in SlowFade™ (ThermoFisher Scientific, #S36936) or VECTASHIELD (Vector Laboratories, #H-1000). Curved retinas were mounted using one or two bridge coverslips to prevent compression.

For brain immunostaining, tissues were dissected in ice-cold Schneider's Medium, fixed for 25 min in 4% PFA, and washed in PBS-T (PBS +1% Triton X-100). Brains were directly incubated in primary solution without prior blocking and processed following the same steps as retinal samples.

Primary antibodies included: mouse Anti-Chaoptin (1:50), rat Anti-Cadherin (1:1000), mouse Anti-En1 (1:100), mouse Anti-Elav (1:50), rabbit Anti-GFP (1:1000), guinea pig Anti-*Dmel*Hth (1:50), guinea pig Anti-*Am*Hth (1:1000), rabbit Anti-*Aa*Hth (1:500), rat Anti-*Aa*Op1(1:50), guinea pig Anti-*Aa*Op8 (1:50), guinea pig Anti-*Dp*Hth (1:100), rat Anti-*Vc*Bar (1:50), rabbit Anti-SalC (1:200), mouse Anti-Rh3 (1:100), and rabbit Anti-Rh6 (1:1000). Secondary antibodies (Alexa Fluor 488, Cy3, or Cy5 conjugates; 1:500) were raised against guinea pig, mouse, rabbit, or rat IgGs.

All samples were imaged using Leica SP8 confocal microscopes equipped with a white laser and HyD detectors. Z-stacks were acquired at 1024x1024x resolution with 0.5 μm with a 20x, 40x, and 63× objective at 400 Hz. Image processing and 3D rendering were performed using Fiji^66^ and IMARIS (v.9.1.2, Bitplane).

#### Behavior assay

Wild type (*Drosophila melanogaster*, Oregon R, isogenized) and *hthΔ675bp* mutant flies were maintained at 25°C and 60% relative humidity under a 12 h light/dark cycle. Three-to four-day-old adult flies were briefly immobilized on ice and mounted on steel pins UV-curable clue, following.^71^ After a 20 min recovery period, flies were placed in a magnetic tethering setup generating a vertical magnetic field with paired permanent magnets.^72^

Polarotactic behavior was recorded under dorsally presented, linearly polarized light with a continuously rotating e-vector (5.97°/s) for 5 min. The polarized stimulus was produced with a custom-built rotating filter cassette,^71^ and flies were illuminated with near-infrared light to avoid visual interference. Recordings were made from below at 60 Hz, and body orientation angles (0–180°) were extracted using Fiji^66^ with a tracking plugin and analyzed in MATLAB (31, MathWorks).

Polarotaxis was defined as periods when the absolute difference between the fly's angular velocity and the rotating e-vector was <3°/s within a 10 s sliding window. Statistical analyses were performed in R (v.4.4.1) using RStudio (v.2024.04.2 + 746). Data normality was tested using the Shapiro-Wilk test, and group comparisons were performed using the Wilcoxon signed-rank test (*p* < 0.05 = ∗).

### Quantification and statistical analysis

#### Quantification of layer targeting

Layer targeting was quantified as in.^69^ Dorsal medulla regions containing DRA and neighboring columns were segmented in IMARIS (v.9.1.2, Bitlane) and analyzed in Fiji.^63^ R7 axon length and ΔR7/R8 distances were measured, and quotient (ΔR7/R8 ÷ R7 length) calculated (*n* = 9 per condition). Data were plotted in GraphPad Prism (v.10.2.2(397)) and compared using unpaired two-tailed t-tests (*p* < 0.05 = ∗).

#### Comparative motif discovery analysis

To examine sequence conservation and identify potential TFBSs within the 675 bp DRA enhancer of *Drosophila melanogaster*, 149 homologous sequences were retrieved by BLAST searches across all *Drosophilidae* genomes available in NCBI (accessed 2021, 69). Sequences were aligned using MAFFT (v7.490, 70) and analyzed with three independent motif discovery and analysis tools: Ciiider (v0.9, 34), XSTREME (v4.11.2, 35), and HOMER (v4.11, 36). TFBM enrichment analyses were performed using two background datasets: (1) intronic regions of *D. melanogaster* homeobox transcription factors, and (2) randomly shuffled input sequences. Enriched motifs were compared against position weight matrices from the FlyFactorSurvey database.^35^

#### Spatial quantification of photoreceptors in DRA across developmental stages

Quantifications were conducted in pupal retinas and adult medulla to assess the DRA photoreceptor distribution and targeting in new and site-mutated reporter lines. Photoreceptors were manually counted in Fiji (v.2.14.0/1.54f; pupal) and IMARIS (v.9.1.2; adult).

In pupal samples, three ROIs – DRA, DA (Dorsal Area) and VR (Ventral Rim) - were defined using Anti-*Dmel*Hth to mark DRA boundaries and Anti-Sal to identify the retinal equator (Figure S5). Sample sized ranges from n = 3–7. Statistical comparisons used unpaired *t* test with Welch's correction (*p* < 0.05 = ∗).

In adult medulla samples, only clearly labeled DRA photoreceptors were analyzed. Group differences among reporter lines were tested by one-way ANOVA (*p* < 0.05 = ∗, *p* < 0.01 = ∗∗, *p* < 0.001 = ∗∗∗).
