# Supplementary material for: Regulatory changes underlying the evolution of skylight navigation
Source: iScience. 2026 Jun 11;29(7):116313. doi: 10.1016/j.isci.2026.116313 (PMC13276312; doi:10.1016/j.isci.2026.116313)
Supplement: Document S1. Figures S1–S5, and Table S1 [file mmc1.pdf]

iScience, Volume *29*

Supplemental information

Regulatory changes underlying

the evolution of skylight navigation

Heidi Roth, Melanie Sarfert, Aleksandra Simdianova, Jana Balke, Michael W. Perry, Katja Nowick, and Mathias F. Wernet

Supplemental Information

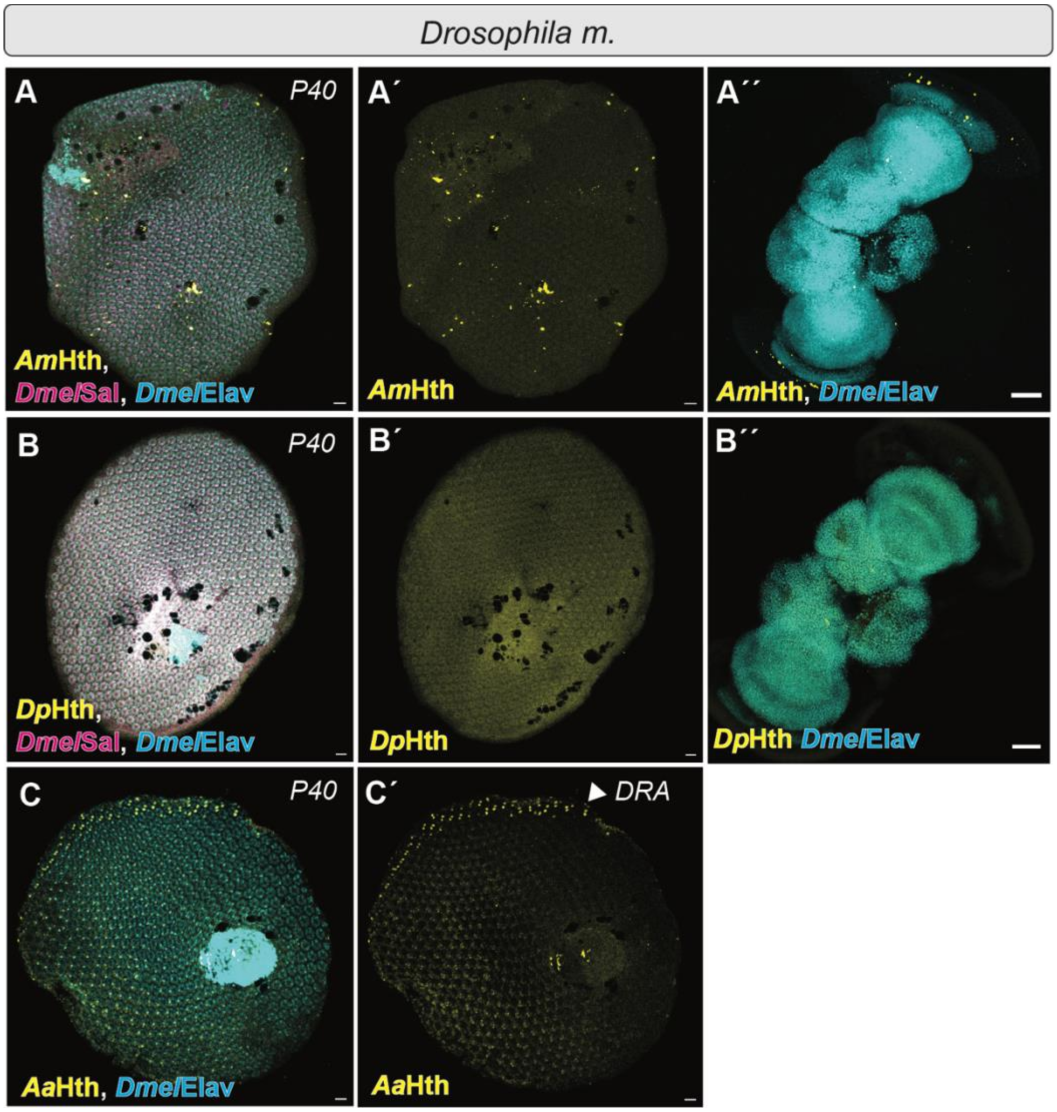

Figure S1: Validation of Anti-Hth antibody specificities for *Drosophila melanogaster*. Wild-type P40 *D. melanogaster* pupal retinas were stained with the respective antibodies from Figure 2. **(C)** Only *Aedes*-derived Anti-Hth antibody specifically labels DRA photoreceptors; honeybee and monarch antibodies show no specific retinal staining and only autofluorescence. No brain staining was detected with honeybee or monarch Anti-Hth antibodies **(A-B´´)**. Per species N = 3 specimens were analyzed. Scale bars: 20 µm.

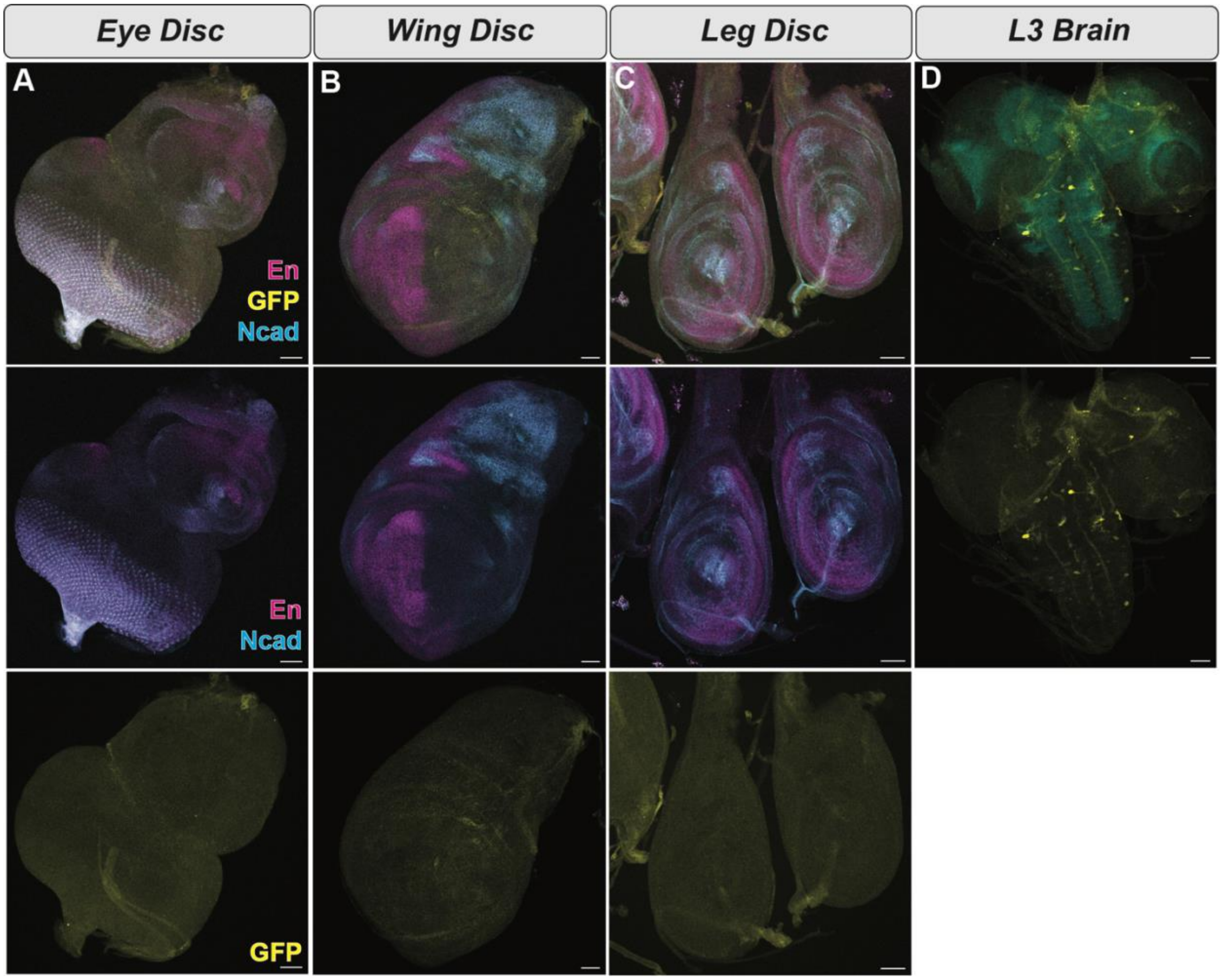

Figure S2: GFP expression driven by 675 bp minimal enhancer during larval development. (A-C) Imaginal discs were co-stained with Anti-Engrailed (*en*, magenta) and an antibody against N-Cadherin (*Ncad*, cyan) as controls, showing no detectable GFP signal driven by the minimal enhancer. **(D)** In larval brains, sparse GFP expression was observed in cells of the ventral nerve cord. N = 3 (larval brains/ per discs type). Scale bars: 50 µm.

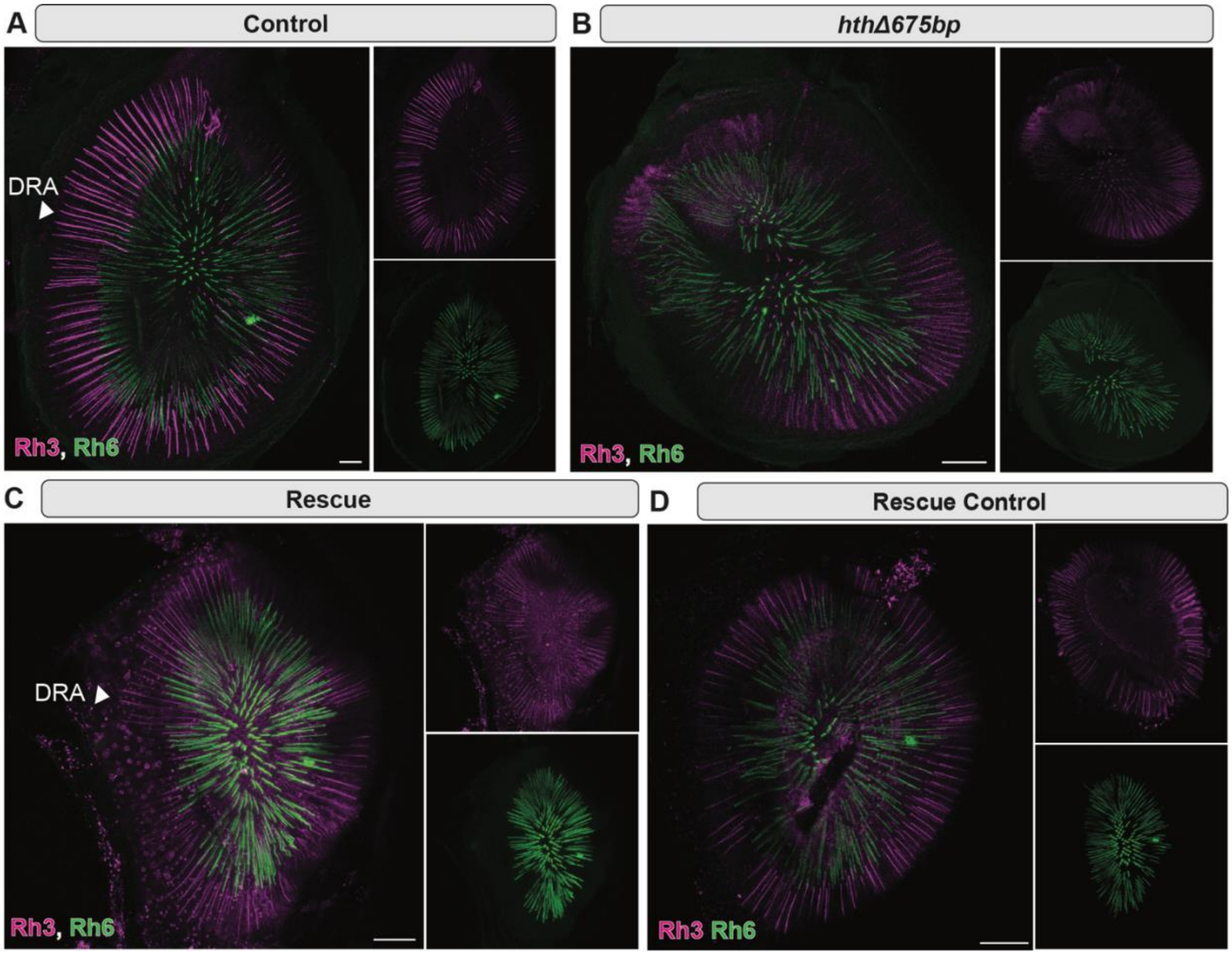

Figure S3: Whole-mounted adult retinas showing rhodopsin expression patterns across conditions. **(A)** Wild-type control retina displaying normal DRA-specific Rh3 (magenta) expression in both DRA R7 and R8, and Rh6 (green) in pale R8 outside of the DRA. **(B)** *hthΔ675bp* mutant retinas showing Rh3 expression limited to R7 and Rh6 in R8 throughout the dorsal region, including former DRA positions. **(C)** Rescue experiment (*hthΔ675bp* in UAS-*Hth* background) restoring Rh3 expression in both DRA R7 and R8. **(D)** Control for rescue experiment displaying the same rhodopsin switch as the mutant (Rh3 in R7 and Rh6 in R8). Scale bar: 50 μm.

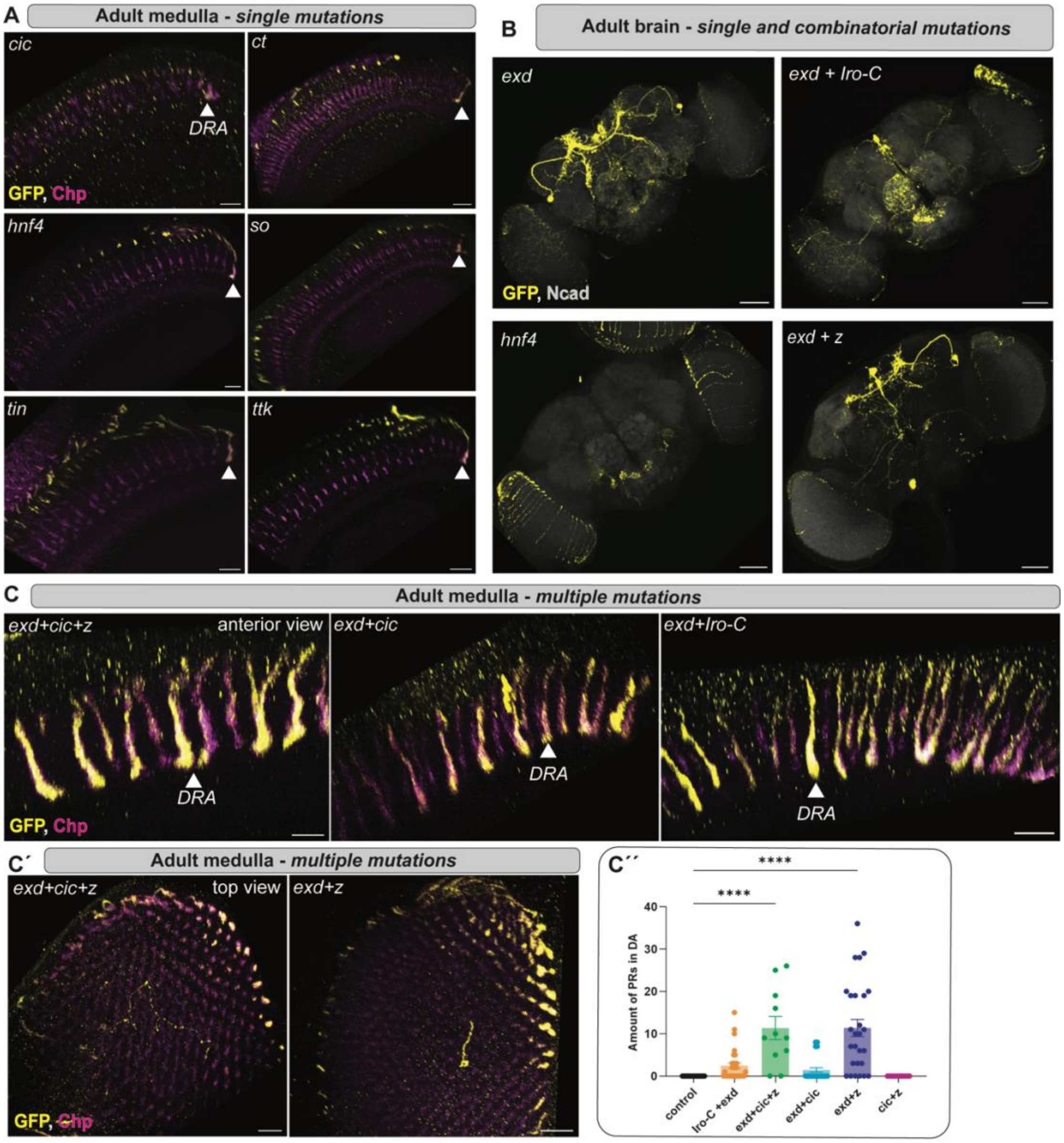

Figure S4: Functional analysis of single and combinatorial TFBS mutations within the 675 bp DRA enhancer. (A) Adult medulla sections showing GFP reporter expression (yellow) and photoreceptors labeling with Anti-Chaoptin (magenta) for remaining single TFBS mutations. All variants exhibit robust GFP expression in DRA columns. **(B)** Adult brain staining of selected single and combinatorial TFBS mutant reporter lines reveal ectopic GFP expression in neurons of the protocerebrum. **(C)** Adult medulla expression patterns of DRA reporter lines carrying combined TFBS mutations. **(C´)** Dorsal view highlighting ectopic GFP-positive photoreceptors in the dorsal area (DA) observed in specific TFBS mutant combinations. **(C´´)** Quantification of GFP-positive photoreceptors located outside DRA columns. Statistical analysis: ordinary one-way ANOVA; significance levels: p<0.05*, p<0.01*, p<0.001***. Error bars: mean±SEM. Scales bars: 50 µm (B), 20 µm (A, C), 10 µm (C´).

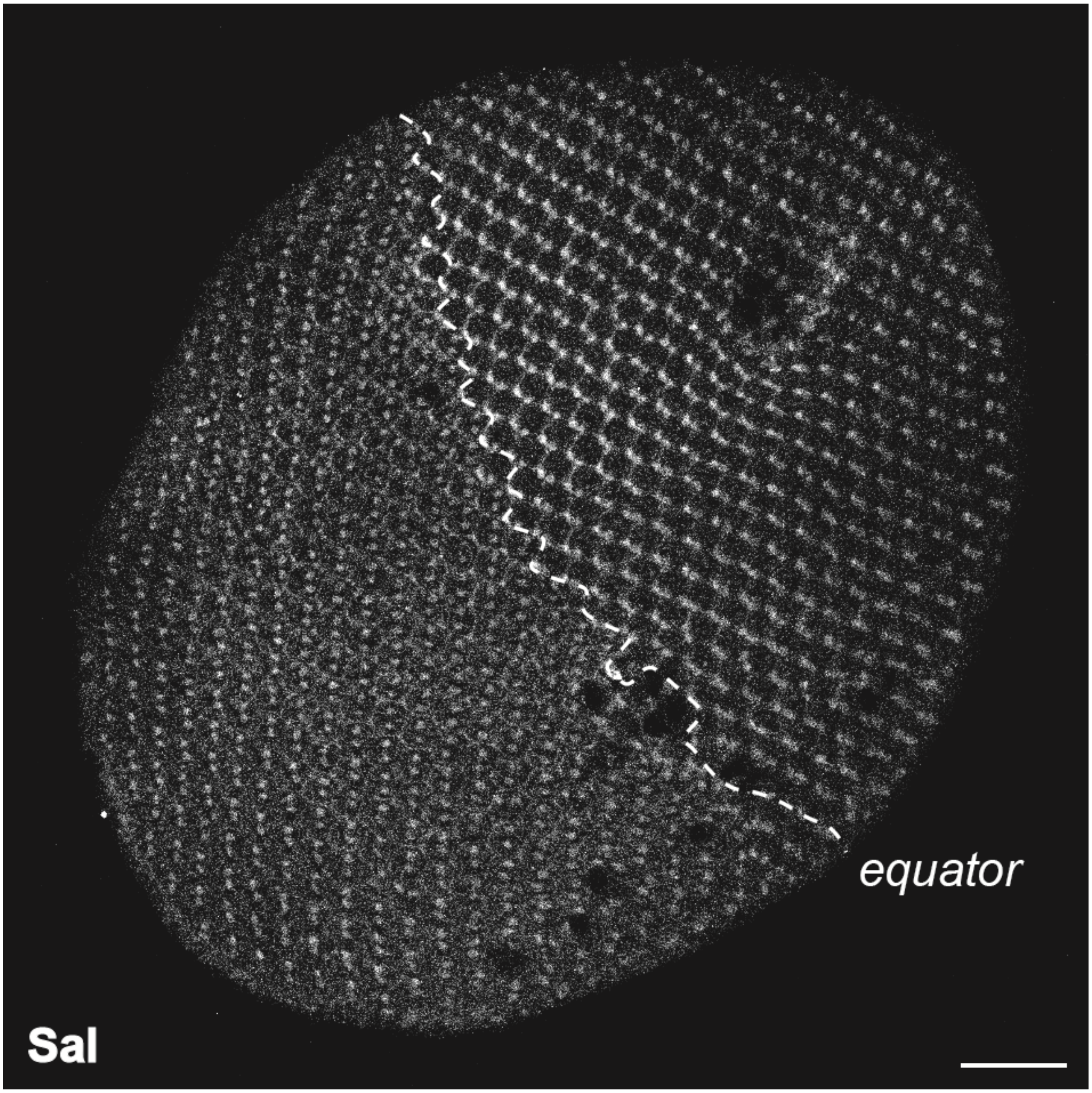

Figure S5: *Drosophila* pupal retina at P40 showing the retinal equator (indicated with dashed line) marked by expression. *Sal* specifically labels R7 and R8 photoreceptors cells in the retina. Scale bar: 20 µm.

Table S1: Genotypes of strains in each figure.

	**Genotypes per Figure**
Figure 1B	CantonS
Figure 2A-A´´´´	Wild type *Apis mellifera* (worker)
Figure 2B-B´´´´	Wild type *Danaus plexippus*
Figure 2C-C´´´´	Wild type *Vanessa cardui*
Figure 2D-D´´´´	Wild type *Aedes aegypti*
Figure 3A	CantonS; wild type *Musca domestica*
Figure 3B-B´´	Wild type *Hermetia illuen*s
Figure 3C-C´´	Wild type *Episyrphus b*
Figure 3D-D´´	Wild type *Ceratitis capitata*
Figure 3E-E´´	Wild type *Lucilia s*
Figure 4A´	yw; UASmCD8::GFP/cyo;UASmCD8::GFP/GMR45D01 Gal4
Figure 4B´	;UASmCD8::GFP/cyo;UASmCD8::GFP/5P Gal4
Figure 4B´	;UASmCD8::GFP/cyo;UASmCD8::GFP/Mid Gal4
Figure 4B´	;UASmCD8::GFP/cyo;UASmCD8::GFP/3P Gal4
Figure 4B´	;UASmCD8::GFP/cyo;UASmCD8::GFP/675bp Gal4
Figure 4D´´	Oregon R (isogenized)
Figure 4D´´´	+;+; *hthΔ675bp/ hthΔ675bp*
Figure 4E	Oregon R (isogenized)
Figure 4E´	+;+; *hthΔ675bp/ hthΔ675bp*
Figure 4E´´	+;GMR45D01Gal4/UAS::Hth; *hthΔ675bp/ hthΔ675bp*
Figure 4E´´´	+;GMR45D01Gal4/cyo; *hthΔ675bp/ hthΔ675bp*
Figure 4F	Oregon R (isogenized, + Ctrl); +;+; *hthΔ675bp/ hthΔ675bp*
Figure 4G	yw; UASmCD8::GFP/Rh6 Gal4; *hthΔ675bp/ hthΔ675bp*
Figure 4G´	yw; UASmCD8::GFP/Rh6 Gal4; *hthΔ675bp/* TM2
Figure 5C	;UASmCD8::GFP/cyo;UASmCD8::GFP/exd Gal4 (kr, Iro-C Gal4)
Figure 5D	;UASmCD8::GFP/cyo;UASmCD8::GFP/ exd+z Gal4 (cic+z Gal4)
Figure 5E	;UASmCD8::GFP/cyo;UASmCD8::GFP/exd Gal4 (cic, ct, exd, Iro-C, kr, so, ttk, z Gal4)
Figure 5F´	;UASmCD8::GFP/cyo;UASmCD8::GFP/exd+cic+z Gal4 (exd+cic, exd+Iro-C, cic+z, exd+z Gal4)
Figure S1 A-C´	CantonS
Figure S2	;UASmCD8::GFP/cyo;UASmCD8::GFP/675bp Gal4
Figure S3	Oregon R (isogenized), +;+; *hthΔ675bp/ hthΔ675bp,* +;GMR45D01Gal4/UAS::Hth; *hthΔ675bp/ hthΔ675bp,* +;GMR45D01Gal4/cyo; *hthΔ675bp/ hthΔ675bp*
Figure S4A	;UASmCD8::GFP/cyo;UASmCD8::GFP/cic Gal4 (ct, hnf4, so, tin, ttk Gal4)
Figure S4B	;UASmCD8::GFP/cyo;UASmCD8::GFP/exd Gal4 (hnf4, exd+Iro-C, exd+z)
Figure S4C-C´	;UASmCD8::GFP/cyo;UASmCD8::GFP/exd+cic+z Gal4 (exd+cic, exd+Iro-C Gal4)
Figure S5	CantonS
